# The PUF binding landscape in metazoan germ cells

**DOI:** 10.1261/rna.055871.116

**Published:** 2016-07

**Authors:** Aman Prasad, Douglas F. Porter, Peggy L. Kroll-Conner, Ipsita Mohanty, Anne R. Ryan, Sarah L. Crittenden, Marvin Wickens, Judith Kimble

**Affiliations:** 1 Department of Biochemistry, and Howard Hughes Medical Institute, University of Wisconsin-Madison, Madison, Wisconsin 53706, USA; 2 Howard Hughes Medical Institute, University of Wisconsin-Madison, Madison, Wisconsin 53706, USA

**Keywords:** FBF, iCLIP, peak-calling methods, target mRNAs, *C. elegans*, PUF proteins, RNA, RNA-binding proteins

## Abstract

PUF (Pumilio/FBF) proteins are RNA-binding proteins and conserved stem cell regulators. The *Caenorhabditis elegans* PUF proteins FBF-1 and FBF-2 (collectively FBF) regulate mRNAs in germ cells. Without FBF, adult germlines lose all stem cells. A major gap in our understanding of PUF proteins, including FBF, is a global view of their binding sites in their native context (i.e., their “binding landscape”). To understand the interactions underlying FBF function, we used iCLIP (individual-nucleotide resolution UV crosslinking and immunoprecipitation) to determine binding landscapes of *C. elegans* FBF-1 and FBF-2 in the germline tissue of intact animals. Multiple iCLIP peak-calling methods were compared to maximize identification of both established FBF binding sites and positive control target mRNAs in our iCLIP data. We discovered that FBF-1 and FBF-2 bind to RNAs through canonical as well as alternate motifs. We also analyzed crosslinking-induced mutations to map binding sites precisely and to identify key nucleotides that may be critical for FBF–RNA interactions. FBF-1 and FBF-2 can bind sites in the 5′UTR, coding region, or 3′UTR, but have a strong bias for the 3′ end of transcripts. FBF-1 and FBF-2 have strongly overlapping target profiles, including mRNAs and noncoding RNAs. From a statistically robust list of 1404 common FBF targets, 847 were previously unknown, 154 were related to cell cycle regulation, three were lincRNAs, and 335 were shared with the human PUF protein PUM2.

## INTRODUCTION

RNA regulatory networks are central to biological control (Keene 2007). Single RNA binding proteins can regulate 100s to 1000s of RNAs with many targets having related functions, which enables coordinated biological control (Keene and Tenenbaum 2002; Gerber et al. 2004; Darnell 2010; Weyn-Vanhentenryck et al. 2014; Hogan et al. 2015; Kershaw et al. 2015; Lapointe et al. 2015; Porter et al. 2015; Wilinski et al. 2015). Understanding RNA regulatory networks in metazoans requires knowing which RNAs are regulated and how they are recognized in their native context. The elucidation of protein–RNA interactions in living tissues and on a global scale is thus a central goal. PUF (Pumilio/FBF) RNA binding proteins are exemplary regulators (Wickens et al. 2002). PUF proteins have been intensely analyzed in yeast, nematodes, flies, and humans at both biochemical and biological levels. They are therefore well poised for studies of RNA regulatory networks.

PUF proteins bind to RNAs via a domain composed of eight three-helical bundles, called Puf repeats (Wang et al. 2001, 2002, 2009; Zhu et al. 2009; Qiu et al. 2012). Most individual Puf repeats recognize a single base in the 7- to 10 nucleotide (nt) long PUF binding element (PBE), although some internal repeats do not make base-specific contacts (Wang et al. 2009; Qiu et al. 2012). PBEs are typically located in mRNAs between the termination codon and poly(A) tail, a region termed the 3′ untranslated region (3′UTR). To date, metazoan PUF binding specificities have been determined using isolated PUF domains (Gerber et al. 2006), truncated PUF proteins in vitro (Bernstein et al. 2005; Opperman et al. 2005; Campbell et al. 2012) or overexpressed PUF proteins in vivo or in tissue culture cells (Galgano et al. 2008; Hafner et al. 2010; Kershner and Kimble 2010). PUF proteins repress their target mRNAs using conserved mechanisms. They can recruit a deadenylase complex, which shortens poly(A)-tail length and destabilizes the transcript (Goldstrohm et al. 2006; Suh et al. 2009), or they can participate in formation of a ternary complex with an Argonaute protein and translation elongation factor to attenuate translational elongation (Friend et al. 2012; Weidmann et al. 2014). In addition to their primary role as mRNA repressors, PUF proteins can also activate mRNA expression or localize mRNAs to control expression spatially (Quenault et al. 2011).

PUF proteins are broadly required for stem cell maintenance, pattern formation, learning, and memory (Lin and Spradling 1997; Spradling et al. 2001; Crittenden et al. 2002; Wickens et al. 2002; Spassov 2004; Salvetti et al. 2005; Kaye et al. 2009; Vessey et al. 2010, 2012; Campbell et al. 2012; Lander et al. 2012). Previous studies of PUF–RNA networks in yeast, nematode germ cells, fly ovaries, mouse testes, and cultured human cells all suggested that PUF proteins are broad-spectrum regulators of the genome, associating with RNAs from 7% to 11% of an organism's genes (Gerber et al. 2004, 2006; Galgano et al. 2008; Morris et al. 2008; Hafner et al. 2010; Kershner and Kimble 2010; Chen et al. 2012; Wilinski et al. 2015). Most of these prior analyses used RIP-chip (RNA immunoprecipitation [IP] followed by microarray analysis of associated mRNAs), which reveals putative target mRNAs but not binding sites within those mRNAs. In contrast, crosslinking and immunoprecipitation (CLIP) identifies both target RNAs (including noncoding RNAs [ncRNA]) and binding sites, and CLIP avoids some background problems inherent to RIP-chip (Mili and Steitz 2004; Moore and Silver 2008). These prior analyses have been valuable and provide a possible outline of PUF–RNA networks. The challenge now is to identify—unambiguously and comprehensively—the RNAs and binding sites for PUF proteins in vivo, in living tissues. As a first step toward that end, we turned to the iCLIP approach to analyze two key stem cell regulators in the nematode germline.

*Caenorhabditis elegans* FBF-1 and FBF-2 (collectively known as FBF) play a major role in maintaining germline stem cells (GSCs) (Crittenden et al. 2002). These paralogs possess 91% identical amino acid sequences, essentially the same binding properties, and are functionally redundant for GSC maintenance (Zhang et al. 1997; Crittenden et al. 2002; Bernstein et al. 2005). Yet FBF-1 and FBF-2 have subtle differences in phenotype and subcellular localization, suggesting they may have distinct targets (Crittenden et al. 2002; Lamont et al. 2004; Voronina et al. 2012). An early RIP-chip study, relying on overexpressed GFP-tagged FBF-1, identified 1350 putative targets (Kershner and Kimble 2010). That work provided information at the gene level as opposed to specific binding sites and suffered from the increased background, decreased specificity, and potential to capture artifactual or indirect interactions, all inherent to RIP-chip (Mili and Steitz 2004; Moore and Silver 2008). Moreover, FBF-1 and FBF-2 offer a powerful model for comparative analysis of iCLIP peak-calling algorithms, because the biochemical basis of their interactions with RNA is well established and a set of positive control mRNAs provide incontrovertible benchmarks. The common peak-calling methods differ considerably based on the CLIP strategy used, available control data sets, and multiple ways to assess variable RNA abundance between genes (Chi et al. 2009; Kishore et al. 2011; Uren et al. 2012; Liu et al. 2015). Comparative data for these methods is scant, possibly because many RNA binding proteins lack validated positive controls necessary for evaluation.

Here we generated iCLIP data for both FBF-1 and FBF-2 from intact animals, investigated the utility of common peak-calling methods, and defined the FBF-1 and FBF-2 binding landscapes. Our findings address major gaps in understanding PUF RNA-binding proteins, including a global analysis of their binding sites and generation of a stringent list of their RNA targets in cells where they normally function.

## RESULTS

### FBF-1 and FBF-2 iCLIP in the germline of an intact animal

**FIGURE 1.**
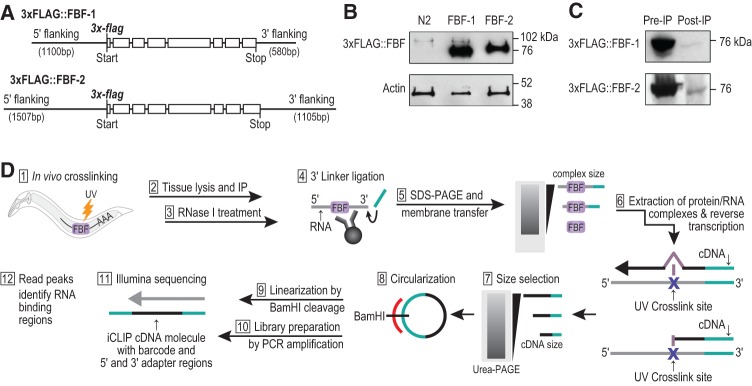
FLAG-tagged FBF-1 and FBF-2 transgenes used for iCLIP in intact animals. (*A*) Transgenes encoding FLAG-tagged FBF-1 and FBF-2. Depicted gene regions possess wild-type *fbf-1* or *fbf-2* sequences with the addition of N-terminal triple FLAG tag. These constructs were incorporated into the *C. elegans* genome as single copies (see Materials and Methods). (*B*) Approximate abundance of FLAG-tagged FBF-1 and FBF-2, assayed by Western blot stained with anti-FLAG antibody. Lysates were prepared from UV crosslinked transgenic animals [FBF-1: *fbf-1(0) 3xflag::fbf-1* and FBF-2: *fbf-2(0) 3xflag::fbf-2*] or from control animals treated identically (wild-type N2). (*C*) Depletion of FLAG-tagged FBF-1 and FBF-2 after IP, assayed by Western blot from transgenic animals as in *B*. (*D*) iCLIP workflow begins with live animals and ends with high-throughput sequencing to elucidate the genome-wide targets of FBF-1 and FBF-2.

We developed reagents to analyze protein–RNA interactions for two key stem cell regulators, FBF-1 and FBF-2. DNAs encoding triple FLAG-tagged FBF-1 or FBF-2 (Fig. 1A) were introduced into the *C. elegans* genome using the MOS Single Copy Insertion technique (Frøkjær-Jensen et al. 2008). We tested the FLAG-tagged FBFs for function by placing them in *fbf-1 fbf-2* double null mutants, which normally lack GSCs and are sterile. Each of the FBF-1 and FBF-2 transgenes rescued *fbf-1 fbf-2* double mutants to fertility (100%; *n* = 20 animals for each transgene). Rescued animals were phenotypically wild-type and FLAG-tagged FBF proteins were enriched in the distal germline, which includes the GSCs (Supplemental Fig. S1), consistent with their endogenous expression patterns (Lamont et al. 2004; Voronina et al. 2012). Also, their expression was essentially limited to the germline (Supplemental Fig. S2). The FBF-1 and FBF-2 transgenes supported a mitotic region of similar size to wild-type and with a normal mitotic index (Supplemental Fig. S3). We conclude that the transgenic FLAG-tagged FBF proteins function appropriately in vivo.

We tested the feasibility of immunopurifying the FLAG-tagged FBFs for use in iCLIP. Genetic crosses were used to generate strains that were homozygous for the transgene and the corresponding null mutant. For example, the transgene harboring N-terminally tagged 3xFLAG::FBF-1 was placed into an *fbf-1* null mutant. Thus, the transgene was the sole source of FBF-1 in the animal. We UV-irradiated ∼50,000 young adults (24 h after the L4 larval stage) to induce crosslinking and immunopurified 3xFLAG::FBF from their lysate (Fig. 1B). 3xFLAG::FBF-1 had a higher mobility than 3xFLAG::FBF-2 in SDS-PAGE, consistent with its smaller molecular weight (73 kDa for tagged FBF-1 versus 75 kDa for tagged FBF-2). The FLAG antibody was specific, as it yielded no signal from wild-type N2 worm lysates. Comparison of pre- and post-IP lysate by Western blots showed nearly complete depletion of the tagged FBFs, demonstrating a highly efficient IP (Fig. 1C). We further confirmed specific enrichment of each FBF protein after immunoprecipitation by Orbitrap mass spectrometry (MS), which resolved specific FBF-1 and FBF-2 peptides at a false discovery rate (FDR) of 1% in IP samples (Supplemental Fig. S4; Supplemental Table S1). Importantly, no peptides from FBF-1 or FBF-2 were detected by MS in wild-type N2 control samples.

We prepared FBF-bound RNA fragments using the published iCLIP protocol (Fig. 1D; Konig et al. 2010; Huppertz et al. 2014). Briefly, living animals were UV-irradiated and then quickly lysed. RNA covalently bound to FBF-1 or FBF-2 was partially digested with RNase I, immunoprecipitated from whole animal lysates with anti-FLAG antibody, and radiolabeled to visualize specific enrichment upon electrophoretic transfer to nitrocellulose membrane and exposure on film. A representative radiolabeling experiment is shown in Supplemental Figure S5. Membrane slabs corresponding to a smear of partially digested, covalently linked FBF–RNA complexes were excised. The RNA was extracted from these membrane pieces. cDNAs were prepared from the RNAs by reverse transcription using randomly barcoded primers, followed by Urea-PAGE size selection, and PCR amplification for Illumina deep sequencing (Fig. 1D). In parallel for each FBF, we prepared negative control libraries from crosslinked wild-type N2 animals by excising the same sized slab of membrane as in the FBF preparations, with regions differing slightly for FBF-1 and FBF-2 due to their different mobilities in SDS–PAGE. Three independent biological replicates were prepared for each FBF and its respective control.

The three biological replicates of FBF-1 iCLIP gave 23,367,131 total uniquely mapped reads (to *C. elegans* genome version WS235) and the three FBF-2 iCLIP replicates gave 14,149,674 uniquely mapped reads (Supplemental Fig. S6). The wild-type N2 samples prepared in parallel to FBF-1 as negative controls yielded 9,596,276 total uniquely mapped reads and those prepared in parallel to FBF-2 yielded 1,092,000 reads. Our finding of variable and sometimes large numbers of reads in negative control data sets is consistent with other CLIP studies (Friedersdorf and Keene 2014) and supports sequencing a set of control samples to fully assess experimental background.

### Selection of an optimal peak-calling method for FBF iCLIP data

FBF-1 and FBF-2 offer a powerful model for selecting an optimal peak-calling method for iCLIP. First, their primary binding motif is established from in vitro experiments. The in vitro FBF binding element (FBE) is UGUNNNAU, where N is any ribonucleotide (Bernstein et al. 2005; Opperman et al. 2005). Moreover, the in vivo relevance of the FBE has been confirmed (Zhang et al. 1997; Merritt and Seydoux 2010). Second, 15 FBF target mRNAs are known to be regulated post-transcriptionally via FBEs (Supplemental Fig. S7) and thus serve as an unusually large number of positive controls compared to other RNA binding proteins. We therefore could evaluate the success of each peak-calling method by considering the number of identified peaks, the enrichment of the FBE, and the recovery of positive control mRNAs as metrics. The most successful method would maximally enrich for the FBE and recover all of the expected mRNAs without dramatically increasing peak number.

**FIGURE 2.**
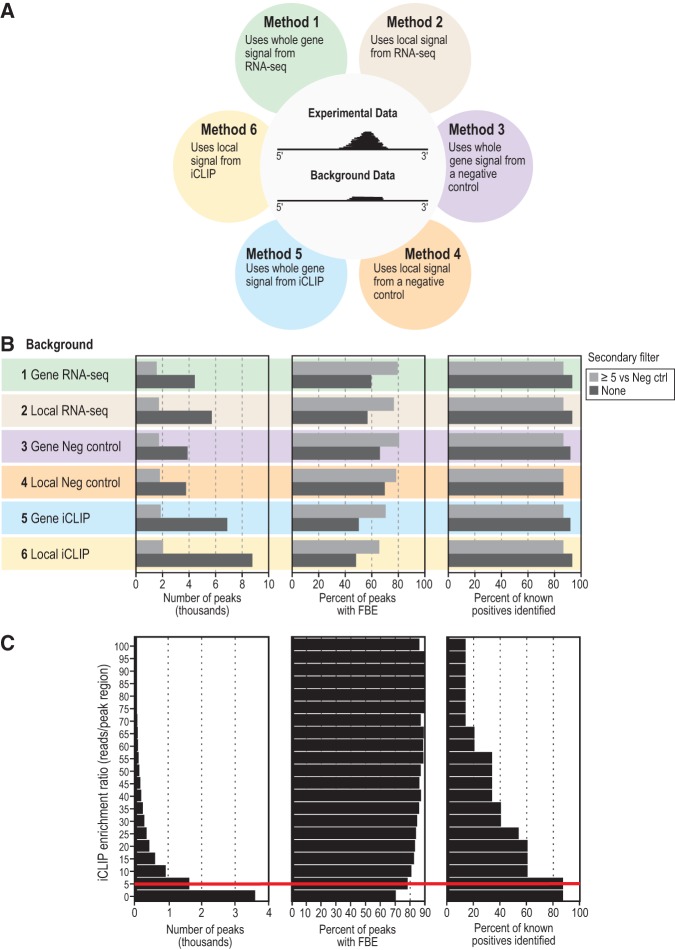
Comparative analysis of six peak calling methods. (*A*) Six methods for peak calling in iCLIP data. Methods were generated using one of three backgrounds, each within one of two genomic regions. The two genomic regions tested were (i) all exons of the target transcript (termed “whole gene signal”) and (ii) the 500-bp genomic region around the putative peak (termed “local signal”). The three types of background coverage were RNA-seq (Methods 1 and 2), parallel iCLIP from a negative control strain (Methods 3 and 4), or the FBF iCLIP data itself (Methods 5 and 6). (*B*) Effects of the six methods of peak calling on number of peaks (*left*), presence of a canonical FBF binding element (FBE) in the peak (*middle*), and the fraction of 15 known FBF target mRNAs correctly identified as targets (*right*). Method numbers and coloring are the same as in Figure 2A. The effect of a secondary filter was also tested, shown as light gray bars. The secondary filter required a minimum enrichment ratio of fivefold more experimental iCLIP reads to negative control iCLIP reads within local (500 bp) peak region. (*C*) Effect of changing the minimum iCLIP enrichment ratio for the secondary filter on the identification of targets. The iCLIP enrichment ratio refers to the ratio of experimental iCLIP reads to negative control iCLIP reads within local (500 bp) peak region. (*Left*) Range of iCLIP enrichment ratios queried. (*Middle*) Percentage of peaks harboring an FBE at each enrichment ratio shown on *left*. Because FBEs are scored in sequences defined by merging peak regions within and between replicates, increasing the filtering ratio can decrease the width of the consensus peak, resulting in the loss of an FBE. (*Right*) Percentage of 15 validated FBF target mRNAs identified at each enrichment ratio shown on *left*. A red line highlights the enrichment ratio cutoff of 5, at which the percentage of peaks harboring an FBE is greatly increased without lowering the number of identified positive control target mRNAs.

We first identified regions of FBF iCLIP reads that had the shape of a peak using parameters that identified read peaks in nearly all positive control mRNAs (Du et al. 2006). We then compared these peak identifications relative to three types of background read coverage in either a 500-bp local region of the genome centered around the peak, or across the mature mRNA (Fig. 2A). Hence, we assessed the use of three background methods within two different regions, yielding six methods (Fig. 2A). The three types of background were RNA-seq data from the MODENCODE project prepared from animals also at the young adult stage (accession: mod Encode_4594) (Method 1 and Method 2), our negative control iCLIP data from wild-type animals (Method 3 and Method 4), or FBF iCLIP signal itself (Method 5 and Method 6). In the latter case (Method 5 and Method 6), a peak is called from the iCLIP signal (and modeled by Poisson) on the assumption that sharp enrichments of signal are true peaks and background will uniformly look like flat coverage. We applied a 1% FDR cutoff to all peaks. Of the methods tested, the most successful (ranked by comparing dark grey bars in Fig. 2B) were those defining background as the negative control iCLIP data from wild-type animals: Methods 3 and 4, which recovered 13 of the 15 positive controls. Two of the 15 positive controls were not in our data by any method. One missing positive, *egl-4*, is an established FBF target in neurons but not the germline (Kaye et al. 2009), which may explain its absence in our data. The second, *fog-3*, is expressed at an earlier developmental stage than that used for our starting material (Chen and Ellis 2000), so its absence was expected. Least successful were Methods 5 and 6, which used FBF iCLIP signal as its own background; also less successful were Methods 1 and 2, which used RNA-seq. We speculate that FBF iCLIP and RNA-seq were less successful as backgrounds because, unlike a negative control iCLIP data set, they did not account for covalently crosslinked background binding inherent to iCLIP.

We also tested the application of a secondary filter to all six methods (lighter shaded bars in Fig. 2B). This filter required a given enrichment ratio of experimental iCLIP reads to negative control iCLIP background reads from wild-type animals within the genomic region spanned by a peak. In all cases, this added filter similarly improved enrichment of the binding site, without losing positive controls. We tested increasingly strict secondary filters (from zero- to fivefold ratios of experimental iCLIP reads to negative control iCLIP from wild-type animals, Fig. 2C). We found that an enrichment cutoff of 5 dramatically raised the percent of peaks containing the binding element, without losing positive controls (red line in Fig. 2C). At higher cutoffs, the gain in FBE enrichment was minimal, but positive controls were lost. We therefore selected a cutoff of fivefold enrichment as a secondary filter.

We conclude that the optimal method to enrich our in vivo iCLIP data for the strongest targets is first to call peaks by comparing to local reads in negative control iCLIP data and then to apply a secondary filter based on a fivefold read enrichment within the local genomic region of the peak (Method 4 in Fig. 2B). We note that this optimized peak-calling method could lose low affinity or noncanonical binding sites given that it is optimized for enrichment of the canonical FBE. However, we still recovered noncanonical binding motifs from our data (see below), suggesting missed sites were not a major limitation of the optimized method. Method 4 was used to assign peaks in all biological replicates of FBF-1 and FBF-2 iCLIP data, and only reproducible peaks were used for subsequent analysis. Specifically, an overlapping peak range must have been called in three out of three biological replicates. Our code for peak assignment is publicly available at https://github.com/dfporter.

We note that an unexpectedly high number of reads was found in the negative control for FBF-1 (∼9 times more than for the negative control for FBF-2). We have no explanation for the high number of control reads for FBF-1, as very similar regions of membrane were excised for the matched controls of FBF-1 and FBF-2. The controls for each were prepared similarly from N2 wild-type worms. Given this issue, we also produced data controlling the FBF-1 data set with the negative control data set for FBF-2. For further analyses, we opted to utilize the data sets where each FBF was controlled with the negative control for FBF-2. In this way, we identified 2345 FBF-1 targets from 2946 peaks and 1457 FBF-2 targets from 1564 peaks. We present all three peak data sets as a resource for future investigations in Supplemental Table S2 (the three data sets being tab 1: FBF-1 versus negative control for FBF-1, tab 2: FBF-1 versus negative control for FBF-2, and tab 3: FBF-2 versus negative control for FBF-2).

Comparison of our FBF RNA targets with a RIP-chip target list previously generated using overexpressed FBF-1 from worm lysate (Kershner and Kimble 2010) found significant overlap (*P*-value <10^−314^, Fisher's exact test), with 41% of 1350 RIP-chip targets present in the FBF-1 and FBF-2 iCLIP target lists. Moreover, a weak but significant correlation (Spearman correlation= 0.23, *P*-value <10^−7^) was found between rank position in our iCLIP lists (ranked by peak height) with rank position on the RIP-chip list (ranked by SAM). Therefore, even though our conditions were much more stringent than those used in RIP-chip, our data conservatively identified many FBF-1 and FBF-2 RNA targets not found previously.

**FIGURE 3.**
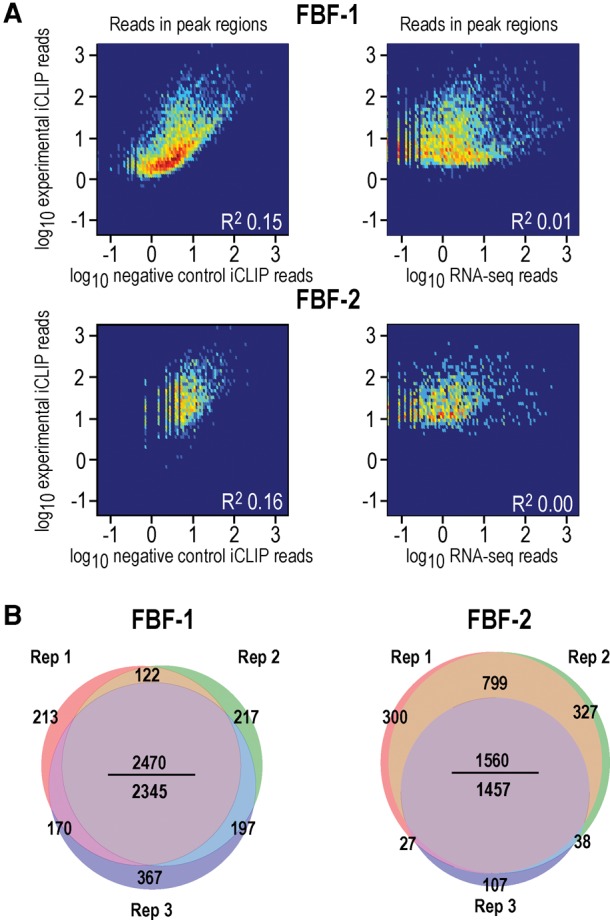
FBF-1 and FBF-2 iCLIP is specific and reproducible. (*A, left*) Heatmaps of FBF iCLIP peak heights (*y*-axis) versus negative control iCLIP peak heights in the same regions (*x*-axis). In these heatmaps, the secondary filter of minimum iCLIP enrichment ratio was not applied, so that the full range of experimental and negative control peaks is visible. Overall, the correlations are low (*R*^2^ = 0.15–0.16), indicating that experimental peaks are distinct from background reads. (*Right*) Heatmaps of FBF iCLIP peak heights (*y*-axis) versus RNA-seq coverage in the same regions (*x*-axis). FBF iCLIP captured binding over a large range of RNA expression levels. Some iCLIP peaks were positively correlated to RNA abundance. This could reflect that peak heights are a function of both binding affinity and RNA abundance (Kishore et al. 2011). (*B*) Venn diagrams depict overlap of targets in three biological replicates of FBF-1 iCLIP (*left*) and three biological replicates of FBF-2 iCLIP (*right*). Targets are highly reproducible. In the *middle* of each Venn, the number *above* the line is total number of overlapping targets in all three replicates and the number *below* the line is the more stringent total number of overlapping targets after requiring that the same peak region on a target be identified in all the replicates. Our analysis used the more stringent *bottom* number.

Several lines of evidence support the validity of our FBF-1 and FBF-2 peak calling. First, read number in putative peak regions correlated poorly with negative control samples (*R*^2^= 0.15–0.16; heatmaps in Fig. 3A, left). Second, correlations between iCLIP peak heights per RNA and RNA-seq read numbers per RNA were low (*R*^2^ ∼0.01). Therefore, the peaks capture protein–RNA interactions across a range of RNA abundances (heatmaps in Fig. 3A, right). We normalized the iCLIP peak heights to RNA abundance using RNA-seq data from two different sources: RNA-seq data from whole worms (MODENCODE) or RNA-seq data from gonads (Ortiz et al. 2014), which afforded tissue specificity. We include raw and normalized peak heights in Supplemental Table S2. Third, targets in the individual biological replicates overlapped well for the FBF-1 and FBF-2 data sets (Fig. 3B), which enables robust statistical analysis. And fourth, for FBF-1, 11 of the 13 positive controls identified were in the top 500 targets ranked by peak height. Similarly, 10 of the 13 were in the top 500 targets for FBF-2. We conclude that our FBF-1 and FBF-2 iCLIP data identify 2345 and 1457 RNAs, respectively, which are enriched for FBF-regulated targets (*P*-value <10^−10^, Fisher's exact test). These iCLIP-derived FBF-1 and FBF-2 target lists likely represent true interactions across a range of binding affinities and were likely not biased by adventitious FBF-RNA interactions.

### FBF-1 and FBF-2 regulate their targets through canonical as well as alternate motif sequences

**FIGURE 4.**
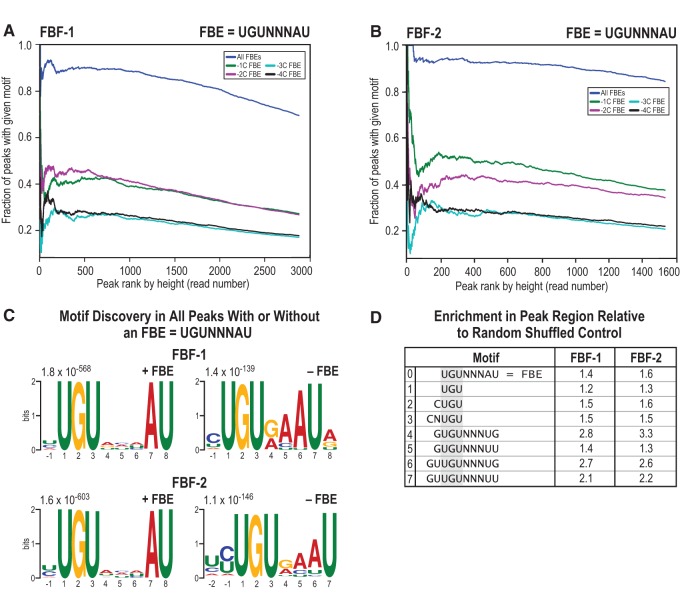
Canonical as well as alternate binding motifs are enriched. (*A*,*B*). Most FBF peaks include the FBF binding element (FBE), and the occurrence of the FBE is proportional to peak height (FBE = UGUNNNAU, where N is any ribonucleotide). FBEs with a −1C or −2C, which confer higher affinity to the binding site, are enriched with opposite preference in FBF-1 and FBF-2. FBEs with an upstream cytosine at increasing distance (−3C and −4C) are less enriched. (*C*) The most significant motifs identified by MEME analysis of FBF peaks, after separating peaks with an FBE from peaks without an FBE. MEME analyses of peaks without an FBE reveal a shorter FBE-like 7-mer sequence. Thirty-one percent of all FBF-1 peaks and 33% of all FBF-2 peaks had a 7-mer. (*D*) Searching for a subset of alternate motifs exclusive of the canonical FBE (which is shown in line 0 in the table) in all peaks reveals significant enrichment (*P* < 0.01) relative to randomly shuffled peak sequences. Lines 1, 2, and 3 represent half-mer sequences based on the UGU trinucleotide characteristic of all PUF protein binding sites. Lines 4, 5, 6, and 7 are motifs identified in an in vitro selection study.

FBF-1 and FBF-2 peak regions in vivo were highly enriched for the canonical FBF binding element defined from in vitro experiments (FBE = UGUNNNAU, where N is any ribonucleotide, dark blue lines in Fig. 4A,B). An upstream cytosine at −1C or −2C relative to the FBE confers especially strong binding in vitro (Campbell et al. 2012; Qiu et al. 2012), and FBF binding sites in the iCLIP data were enriched for these high-affinity FBEs (green and magenta lines in Fig. 4A,B). Interestingly, for the highest ranked targets, FBF-1 was more enriched for −2C FBEs, and FBF-2 was more enriched for −1C FBEs, suggesting differential in vivo preference for either a −1C or −2C. Cytosine nucleotides increasingly distant from the FBE were not enriched (−3C and −4C, light blue and black lines in Fig. 4A,B). A more detailed look at every nucleotide in the binding site agrees with previous work on nucleotide composition of the canonical FBE (Bernstein et al. 2005; Opperman et al. 2005), but we observed that a G or A was favored after the final U (Supplemental Fig. S8A). Considering enrichment at every position of the canonical motif, including flanking positions, the overall consensus motif we obtained in vivo could therefore best be described as a −1 or −2 C followed by UGURCCAUR. We note that the strongest FBE in the positive control target *gld-1* (CAUGUGCCAUA) (Crittenden et al. 2002), through which FBF maintains GSCs, is a perfect match to the consensus we derived, while the FBE in *fem-3* (CUUGUGUCAUU) (Ahringer and Kimble 1991) is suboptimal in the two underlined nucleotides.

We noted the lack of a canonical FBE in 31% of the peaks called for FBF-1 and 16% of the peaks called for FBF-2 and so we asked if motifs other than the canonical FBE were bound in vivo. Indeed, many CLIP studies find a large fraction of peaks without the canonical binding element (Darnell 2010). In order to identify how FBF recognizes RNA regions without an FBE, we separated peaks into two sets based on the presence or absence of the canonical FBE and performed motif analysis on both sets of peaks.

Intriguingly, in peaks without the FBE, the most significant motif identified by MEME was a shorter FBE-like 7-mer sequence with decreased internal degeneracy (Fig. 4C). After reanalyzing all peaks for occurrence of this 7-mer, we found that 31% of FBF-1 peaks and 33% of FBF-2 peaks had the 7-mer. Careful analysis of the 7-mer motif identifies an optimal sequence of a -1 or -2 C, followed by UGUGAAUR (Supplemental Fig. S8B). In a previous study, FBF-1 was found to bind an FBE-like sequence that is short by 1 nt in the middle degenerate positions (UGUGCAUA), both in yeast three-hybrid and gel shift assays, albeit at ∼20-fold lower affinity than canonical sites (Opperman et al. 2005). In our data, the median peak coverage was 1964 reads over a canonical FBE and 1620 at the shorter 7-mer sequence. This difference is significant (*P* < 0.01, *t*-test), consistent with a lower preference and possibly lower affinity for the 7-mer element. One caveat, however, is that peak height is not a direct measurement of binding affinity because it also reflects abundance of the specific RNA.

Motifs other than the canonical FBE or the 7-mer were also found in FBF in vivo-bound peaks. MEME identified “half-mer” sequences, such as UGU and CUGU, as enriched in peaks without an FBE. In addition, previous in vitro analysis had identified four noncanonical U- and G-enriched FBF binding motifs (Campbell et al. 2012), and we asked if these in vitro*-*derived motifs were bound by FBF in vivo. We therefore analyzed all peak regions for enrichment of seven noncanonical motifs, including those identified in our data or suggested from other studies (motifs 1 through 7 in Fig. 4D). The canonical FBE, motif 0, is provided for reference. Motifs 1, 2, and 3 represent half-mer sequences, with or without upstream cytosine and based on the UGU trinucleotide characteristic of all PUF protein binding sites. Motifs 4, 5, 6, and 7 are motifs identified in an in vitro selection study (Campbell et al. 2012). Here, our analysis excluded canonical FBEs if one or more was present in the peak. Seven of the eight noncanonical motifs were enriched (Fig. 4D). Motifs 4 through 7 were important for FBF binding in vitro when assayed together with the FBF protein interactor CPB-1 (Campbell et al. 2012). Our data suggest that these in vitro-derived motifs may be bound more generally, because CPB-1 is not abundant in adults (Luitjens et al. 2000), the stage assayed here. Interestingly, the in vitro-derived motifs 4 through 7 diverge from the canonical motif in having enrichment of upstream and downstream G and/or U nucleotides. We also considered enrichment of UGUGUAUAUA, a motif important for FBF binding of *egl-4* mRNA (Kaye et al. 2009); *egl-4* is a target in neuronal tissue but not identified as a target in our study. We did not find enrichment of the UGUGUAUAUA *egl-4* motif in our data. Taken together, the enrichment of motifs other than the FBE, hereafter termed “alternate motifs,” suggests previously unknown binding modes for FBF-1 and FBF-2 in vivo.

### Crosslinking-induced mutations and truncations precisely mark FBF-1 and FBF-2 binding sites

**FIGURE 5.**
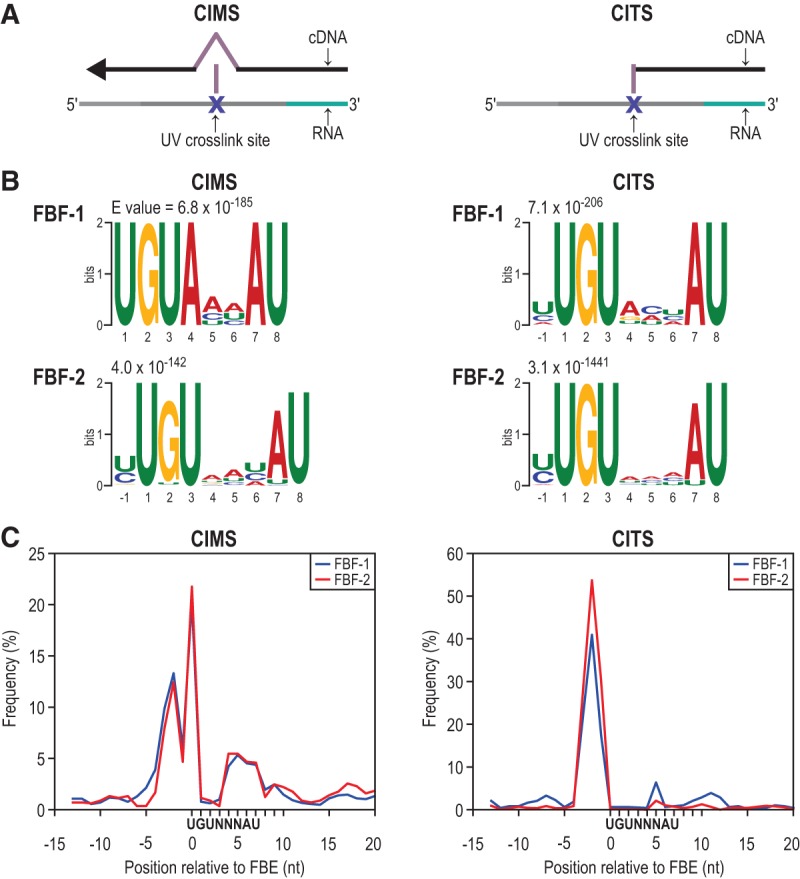
Mapping FBF-1 and FBF-2-RNA crosslink sites. (*A*) Diagrams of CIMS analysis (*left*) and CITS analysis (*right*). Both identify protein:RNA crosslink sites by detecting errors made in reverse transcription during cDNA library preparation. (*B*) The most significant motifs identified by MEME analysis in a 21-nt window centered around significant (*P* < 0.001) CIMS and CITS sites are the FBEs (UGUNNNAU, where N is any ribonucleotide). There is a variable nucleotide preference at the −1 and 4, 5, and 6 positions. (*C*) Crosslink site enrichment relative to the FBE for all FBE-containing clusters demonstrates FBF crosslinks predominantly upstream of the FBE.

We next sought to learn at single-nucleotide resolution where FBF crosslinks within each peak. Two analysis pipelines have been used previously for this purpose: crosslinking-induced mutation site (CIMS) analysis and crosslinking-induced truncation site (CITS) analysis (Zhang and Darnell 2011; Weyn-Vanhentenryck et al. 2014). Both CIMS and CITS take advantage of events caused during reverse transcription for cDNA library preparation (Fig. 5A). CIMS identifies amino acids crosslinked to RNA as insertions, deletions, or substitutions, whereas CITS identifies them as truncations. One advantage of iCLIP is that it can recover both CIMS and CITS errors in a single experiment, compared to just CIMS errors. In CLIP experiments, truncation occurs more often than read-through errors (Sugimoto et al. 2012), and UV crosslinking induces deletions almost exclusively among the various read-through error types (Moore et al. 2014). We applied CIMS and CITS analysis to the FBF iCLIP data and considered only reproducible, significant crosslink sites (*P* < 0.001).

We identified 6136 CIMS loci and 1019 CITS loci reproducibly crosslinked to FBF-1 and 2570 CIMS loci and 2592 CITS loci reproducibly crosslinked to FBF-2. We first searched for motifs in a 21-nt window around these CIMS and CITS sites using MEME (Bailey et al. 2009), as this could both validate our crosslink identifications if we enriched for the FBE and provide a more precise map of RNA-contact points than searching the entire peak. From this search, we uncovered the FBE as the most significant motif (Fig. 5B). We also observed enrichment at the −1 position for either a C or U (Fig. 5B), which suggests an effect of these residues on binding affinity. A C at −1 was shown previously to enhance binding (Qiu et al. 2012), but a U has not been reported to increase affinity. Its enrichment here could indicate that an upstream U enhances binding affinity or it could indicate that there is a bias in our data for crosslinking to U bases, which can occur in CLIP experiments (Sugimoto et al. 2012).

We then derived a positional map of crosslink site enrichment relative to FBEs (Fig. 5C). We found that nucleotides immediately upstream of the FBE were the primary crosslinking sites, but crosslinking was also enriched at U1 and the 3′-most four nucleotides of the FBE. Therefore, FBF directly contacts nucleotides in and around the FBE, with the primary crosslinking site being an extended sequence immediately upstream of the target motif. Other CLIP studies have also found crosslink enrichment in regions adjacent to binding elements: for example, PARCLIP of human PUM2 in cell culture (Hafner et al. 2010) and HITS-CLIP of Nova in mouse brain (Licatalosi et al. 2008). Our data indicate that nearby upstream and downstream residues outside of the binding element can influence FBF–RNA interactions. Moreover, we found crosslinking most enriched near the UGU trinucleotide of the motif (Fig. 5C) as opposed to the downstream end of the motif, which is the less conserved end of PUF binding elements within the PUF family.

### FBF-1 and FBF-2 have similar binding landscapes

We asked if FBF-1 and FBF-2 bind the same RNAs in vivo as a way of investigating whether their molecular functions are comparable. With our optimized peak caller that included a read enrichment cutoff, we obtained 888 more FBF-1 targets than FBF-2 targets (Fig. 3B). One possibility is that FBF-1 truly binds many more targets than FBF-2. However, we suspect that more FBF-1 targets were found because the FBF-1 data set was more complex (Supplemental Fig. S6). Ninety-seven percent of FBF-2 called peaks overlapped with FBF-1 peaks and all the excess FBF-1 targets also had some signal in the FBF-2 data.

**FIGURE 6.**
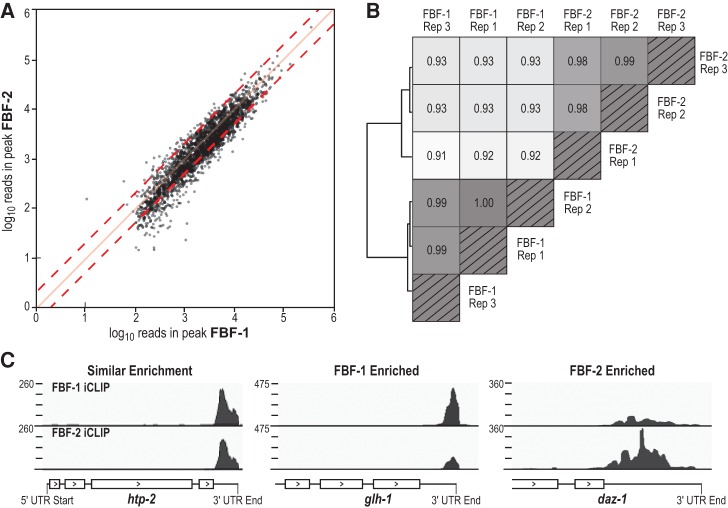
FBF-1 and FBF-2 have highly similar binding landscapes. (*A*) FBF-1 and FBF-2 peak heights and peak regions are highly correlated. The FBF-2 data set was normalized to the size of the FBF-1 data set. Red dashed lines mark twofold enrichment and solid red line marks a slope of one. (*B*) Hierarchal clustering of correlations between and within FBF-1 and FBF-2 replicates. (*C*) Snapshots of enrichment differences for select targets. Coverage is given in reads per million, with the coverage range shown in the *upper left*.

In addition to FBF-1 and FBF-2 binding a similar set of RNA targets, their peak heights and locations were also highly correlated (Pearson *R* = 0.82, Fig. 6A). However, the two data sets did have quantitative differences. When replicates were clustered by their Spearman correlations, FBF-1 replicates were more similar to each other than to FBF-2 replicates (Fig. 6B). This likely reflects statistically significant differences in many of the individual peak heights between the two data sets (Supplemental Table S3). For example, the 10 most enriched mRNAs for FBF-1 (in descending order of fold enrichment) were *far-3*, *ZK484.5*, *F12A10.1*, *F14H3.5*, *spr-1*, *R07E4.5*, *nkat-3*, *Y53F4B.42*, *meg-1*, and *sec-3*; the 10 most enriched mRNAs for FBF-2 were *Y54E2A.4*, *vab-1*, *C16C8.11*, *uaf-2*, *W09C5.1*, *daz-1*, *pink-1*, *W09C5.7*, *Y51H4.13*, and *C56C10.11*. Specific examples of differential target enrichment are shown in Figure 6C (*glh-1* for FBF-1 and *daz-1* for FBF-2, with *htp-2* shown as a reference for similar enrichment). While it remains possible that FBF-1 and FBF-2 bind unique RNAs or that our failure to identify unique targets may be technical, we favor instead the idea that FBF-1 and FBF-2 bind essentially the same RNAs at the same sites. This idea is consistent with their biological redundancy in stem cell control, but not with minor phenotypic differences, which we do not yet understand. By MEME analysis, we could not detect significant differences in motifs within specifically enriched peak regions, reinforcing the strong similarity of the FBF-1 and FBF-2 binding landscapes.

Given the similarity of the FBF-1 and FBF-2 target lists, we took advantage of our multiple biological replicates to create a statistically strong “FBF” data set that assigned targets based on whether the same peak was detected in at least five out of the six FBF-1 plus FBF-2 replicates. This strategy identified 1609 “FBF peaks” in 1404 target RNAs (Supplemental Table S2, tab 4). All known targets (13/13) were among the top 700 targets in this combined FBF list, and the most enriched motifs by MEME analysis were the canonical FBE (UGUNNNAU, *E*-value = 10^−278^) and, in peaks without an FBE, the 7-mer sequence (UGURNAU, *E* value = 10^−130^). This combined FBF list provides a robust genomic profile of FBF binding for further analyses. Henceforth, we call the peaks and targets gleaned by combining FBF-1 and FBF-2 data the “FBF peaks” and “FBF targets” for simplicity.

### Genome-wide map of FBF binding reveals location-dependent characteristics of PUF regulation in vivo

**FIGURE 7.**
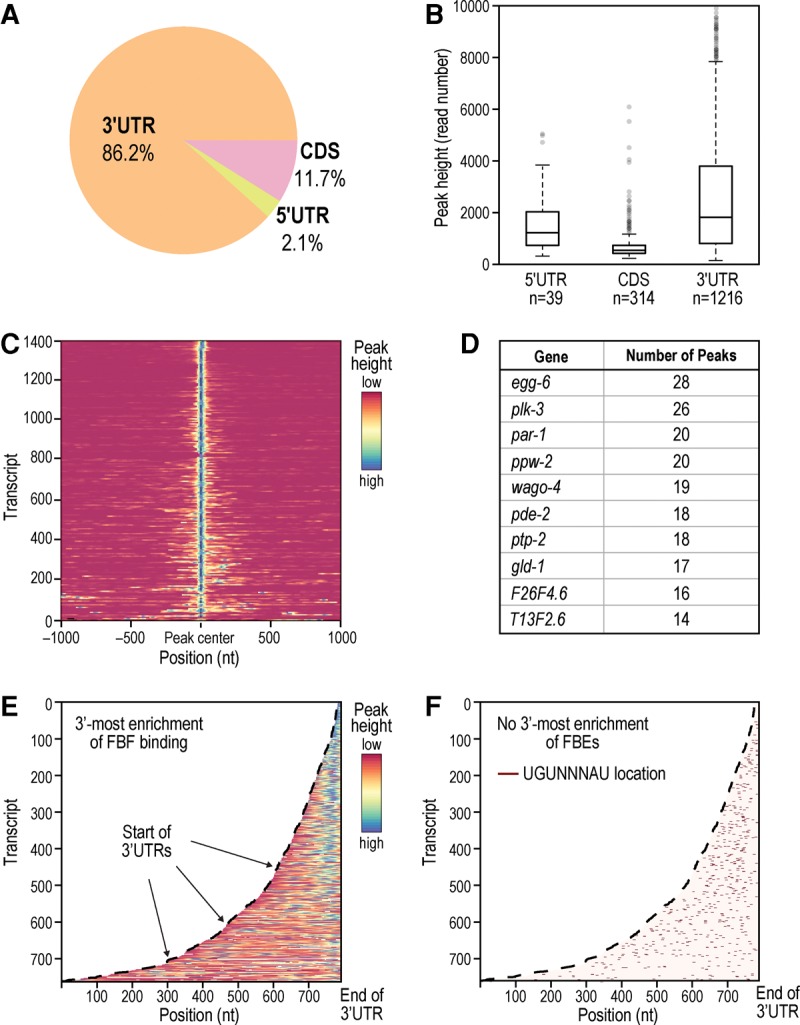
Binding locations of FBF. (*A*) FBF primarily binds in 3′UTRs, but other binding locations are utilized. Only the tallest peak in a gene was included in this pie chart. (*B*) 3′UTRs also have the highest average peak height among binding locations. All 1569 peaks in mRNA are included. (*C*) Heatmap of peak signal within target transcripts spanning 1000 bp on either side of the primary (highest) peak. A fraction of targets (especially visible toward the *bottom* of the heatmap) has multiple peaks at a variable distance to the primary peak. (*D*) Top 10 targets with the most detected peaks. (*E*,*F*) Heatmaps of peak signal in 3′UTRs (*E*) and canonical FBE distribution (*F*). For all peaks, FBF binding is biased toward the 3′-most end while FBEs are more scattered.

We mapped the locations of peaks within the 1404 FBF targets. Binding was highly enriched in 3′UTRs, but also found in 5′UTRs and coding regions (Fig. 7A; Supplemental Fig. S9A). Average peak heights were greater in the 3′UTR than in either 5′UTR or coding sequence (Fig. 7B). FBF binding is therefore highly biased toward binding sites in the 3′UTR.

The expanded repertoire of FBF binding locations is consistent with findings from CLIP studies of other RNA binding proteins from diverse organisms, including FMRP in mouse brain and GLD-1 in *C. elegans* (Chi et al. 2009; Darnell 2010; Hafner et al. 2010; Jungkamp et al. 2011; Anko et al. 2012). Excluding 3′UTR sites, 38 targets contained at least one 5′UTR peak (listed in Supplemental Table S4, tab 1), and 265 targets contained at least one CDS peak (Supplemental Table S4, tab 2). With respect to peaks in multiple locations, 12 targets contained both 3′UTR and 5′UTR peaks (Supplemental Table S4, tab 3), 110 targets contained 3′UTR and CDS peaks (Supplemental Table S4, tab 4), and 6 targets contained peaks in all three locations (Supplemental Table S4, tab 5). Single 5′UTR binding sites close to the start codon or dual 3′-and-5′UTR sites can affect translational regulation (Jungkamp et al. 2011). Representative snapshots of FBF 5′UTR binding are shown in Supplemental Figure S10 for three of the 38 targets, with distance to the start codon denoted. Among the 38 FBF targets with 5′UTR peaks, three have established roles in regulating metazoan stem cells (*ife-1, fbf-1,* and *fbf-2*). *ife-1* encodes a germline-specific eIF4E translation factor and promotes GSC differentiation (Henderson et al. 2009). The 3′UTR peaks in *fbf-1* and *fbf-2* mRNAs are consistent with autoregulation, as seen for other RNA binding proteins including other PUF proteins (Lamont et al. 2004; Dredge et al. 2005; Buratti and Baralle 2011; Anko et al. 2012; Weidmann and Goldstrohm 2012; Jangi et al. 2014).

In addition to possessing a primary (highest) binding peak, 11% of the FBF targets (156 genes) contained one or more secondary peaks (heat-map visualization of peak signal in Fig. 7C). For this analysis, we reapplied the peak finding algorithm of Du et al. (2006) to “un-merge” merged peaks and count all distinct interaction sites separately. The 10 targets with the most peaks encode several key germline regulators, including GSC regulators (Fig. 7D). Overall, primary peaks had a characteristic shape: a sharp, nearly symmetrical region ∼40 nt wide (Supplemental Fig. S11). Secondary peaks were less sharp and were biased to a region upstream of the primary peaks. 81% of primary peaks contained a canonical FBE whereas 46% of secondary peaks contained a canonical FBE. Together, our results suggest that FBF binding generally protects a roughly 40-nt region around the canonical FBE, but we also discovered additional FBF contacts (i.e., secondary peaks), mostly upstream. We also found that multiple peaks were in longer transcripts (median mature transcript length of 2.4 kb for transcripts with multiple peaks versus 1.7 kb for transcripts without multiple peaks), which was significant (*P* < 0.05). Multiple peaks may represent multiple FBFs simultaneously bound to a single transcript, perhaps to enhance repression of longer transcripts. For example, multiple peaks on targets of the RBP hnRNP C mark ribonucleoprotein complex formation from multiple hnRNP C subunits (Konig et al. 2010). However, our data were obtained from a population, so multiple peaks may also represent a population of single binding events.

We found that FBF binds at the 3′-most end of its targets. Raw peak signal visualized in 3′UTRs (Fig. 7E) is highest at the end of the target sequence. Peak signal becomes more dispersed in longer 3′UTRs (>200 bp). The 3′ bias is not explained simply by FBE location, as canonical FBEs in the same set of transcripts are more randomly dispersed (Fig. 7F), and read coverage of 3′UTR FBEs is positively correlated with a closer proximity of the FBE to the 3′ end of the transcript (Supplemental Fig. S9C, *P*-value <10^−7^, Spearman). This suggests FBF prefers the terminal-most FBEs. Enrichment near the 3′ end of transcripts is also apparent when viewing signal in the entire RNA (Supplemental Fig. S9A), a greater enrichment than observed for the FBE (comparison of Supplemental Fig. S9A,B). FBF enrichment at 3′ ends of 3′UTRs is consistent with the conserved PUF mechanism of mRNA repression—recruitment of the Not complex and deadenylation of their targets (Goldstrohm et al. 2006; Kadyrova et al. 2007; Goldstrohm and Wickens 2008; Suh et al. 2009; Van Etten et al. 2012).

### FBF targets

We focused on the 1404 stringently defined FBF target RNAs to analyze their coding content. This robust list includes 557 RNAs previously identified using RIP-chip (Kershner and Kimble 2010), plus an additional 847 not previously found. Using gene ontology (GO) analysis (Huang da et al. 2009a,b), we evaluated three categories: biological process, cell component and molecular function. The most enriched biological process was the cell cycle, with nearly 11% in that category (154/1404); that 11% included key checkpoint regulators and core machinery for both mitotic and meiotic divisions, reflecting broad control of the cell cycle (Supplemental Fig. S12A). The most enriched cell components were microtubule or chromatin associated (Supplemental Fig. S12B), likely reflecting cell cycle enrichment. The most enriched molecular function was nucleotide binding (Supplemental Fig. S12C) with many RNA binding proteins, including seven PUF proteins (*fbf-1*, *fbf-2, puf-5, puf-6, puf-7, puf-8,* and *puf-10*), three CPEB proteins (*cpb-1*, *cpb-3*, and *fog-1*), three germline helicases (*glh-1, glh-2,* and *glh-3*), two splicing regulators (*rsp-2* and *swp-1*) and other uncharacterized genes with predicted RNA binding function. We conclude that FBF is a major regulator of the cell cycle and other RNA-binding proteins, and that it also participates in autoregulation (Lamont et al. 2004) and PUF cross-regulation.

Beyond mitotic and meiotic regulators, we found key components from a spectrum of developmental, signaling, and RNA regulatory pathways, all of which are critical for proper germ cell function (Kimble and Seidel 2013; Schedl 2013). In this vein, our iCLIP results found several pathways first identified by FBF-1 RIP-chip (Kershner and Kimble 2010), confirming certain components of those pathways, but also extending the number of pathway components under FBF regulation. For example, iCLIP found 18 genes critical for germline sex determination (Supplemental Table S5, tab 1), 7 of which were not identified previously (*ddx-23, fem-1, daz-1, gls-1, larp-1, puf-8,* and *tra-1*). We also confirmed and extended FBF's binding to mRNAs that encode major components of Notch, MAP kinase, and Wnt/β-catenin signaling. Finally, the iCLIP data revealed many factors critical for RNA regulation. Thus, FBF binds multiple mRNAs encoding parts of the deadenylation machinery (*let-711/ntl-1, ntl-9, panl-2, panl-3*), the 5′ to 3′ decay pathway (*cgh-1, lsm-1, lsm-7,* and *xrn-1*) as well as essential machinery for miRNA biogenesis and miRNA-mediated gene silencing (*dcr-1, drh-3,* and the Argonauts *ergo-1, prg-1, prg-2, ppw-2, wago-2, wago-4* and *C14B1.7*). Given that FBF regulates mRNAs by recruitment of the deadenylation machinery and components of the miRNA processing pathway (Suh et al. 2009; Friend et al. 2012), its repression of the same machinery may buffer its activity.

Since the PUF domain is highly conserved, we assessed target overlaps of cytoplasmic PUFs from *S. cerevisiae* (Puf3p and Puf5p [Lapointe et al. 2015; Wilinski et al. 2015]), nematode (FBF, this work), and human (PUM2 [Hafner et al. 2010]). In this analysis, we considered only high resolution data sets derived from “RNA Tagging” or CLIP experiments. We found that 286 of our 1404 FBF targets are shared with 1418 *S. cerevisiae* Puf3p or Puf5p targets (Supplemental Table S5, tab 2, *P*-value <0.01 for overlap by Fisher's exact test). 335 FBF targets are shared with 2579 PUM2 targets (Supplemental Table S5, tab 3, *P*-value <0.01 for overlap by Fisher's exact test; Hafner et al. 2010), and 84 targets are shared by yeast, worm, and human PUFs (Supplemental Table S5, tab 4, *P*-value <0.01 for overlap by Fisher's exact test). Interestingly, among these 84 shared targets is *ccf-1*/POP2, a 3′ to 5′ exoribonuclease that is part of the Ccr4-Not deadenylase complex important for mRNA degradation (Nousch et al. 2013). We conclude that some PUF targets are conserved among highly divergent organisms. Overall, 793 of the 1404 FBF targets have a human orthologue (Supplemental Table S5, tab 5; Shaye and Greenwald 2011). Of these 793 targets, 71 are present among the 154 cell cycle related FBF targets, and 35 of the 71 are also PUM2 targets (Supplemental Table S5, tab 6), indicating FBF binds conserved cell cycle regulators that are also targeted by a human PUF.

**FIGURE 8.**
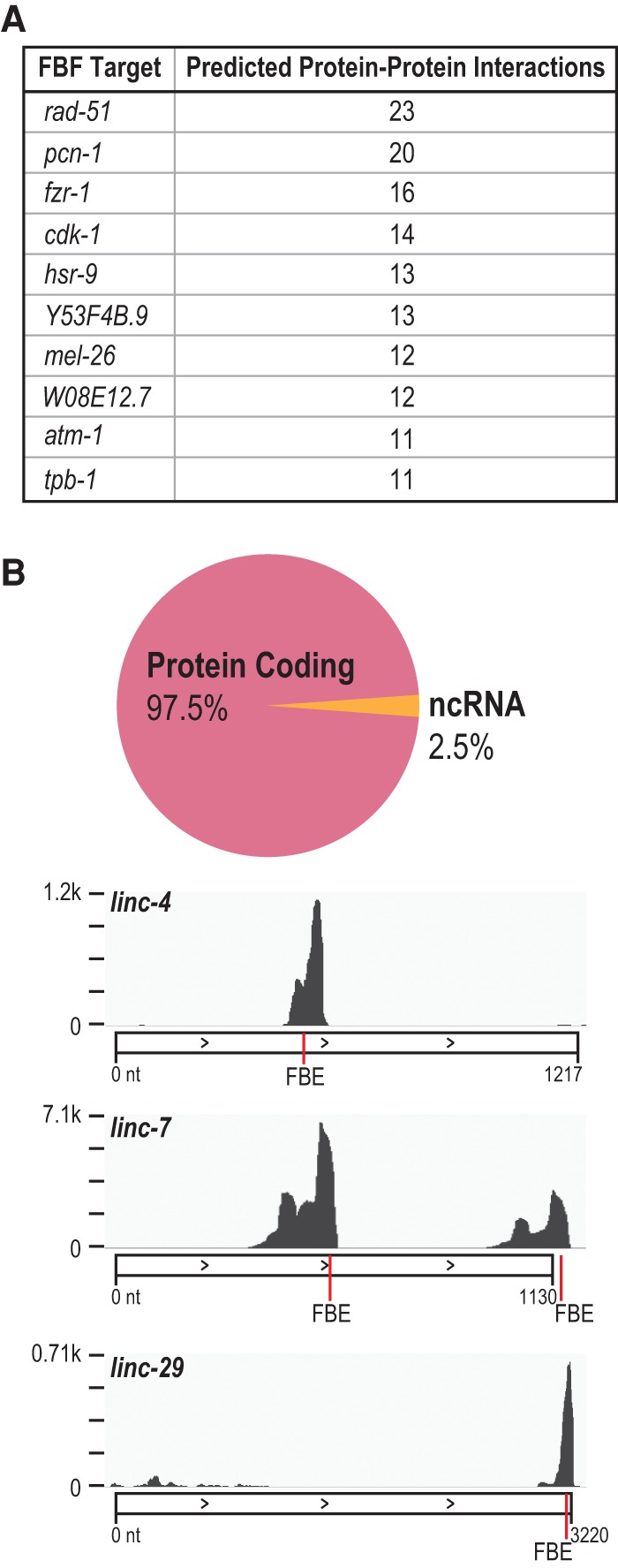
FBF targets. (*A*) Top 10 targets that have predicted interactions with other FBF targets by STRING-DB analysis at a high confidence setting. These targets may be hubs in the large FBF-regulated RNA network. (*B*) FBF primarily targets protein-coding genes but also a small fraction of noncoding RNA (ncRNA). Snapshots of FBF binding to long noncoding RNA (lincRNA) targets are shown, with the combined FBF-1 and FBF-2 raw coverage depth on the *y*-axis. FBE locations in the peak marked in red.

We found that FBF binds predominantly to protein coding mRNAs (Fig. 8B). However, several noncoding RNAs (ncRNAs) were also bound. Peaks with canonical FBEs located in ncRNAs identified three long intervening ncRNAs (lincRNAs) of unknown function – *linc-4, linc-7,* and *linc-29* (Fig. 8B). *linc-7* had multiple binding sites. Each is supported as a noncoding RNA by several lines of evidence (discussed in Nam and Bartel (2012), including ribosome profiling that is negative for ribosome occupancy. The three lincRNAs are not related to each other and are not conserved in humans. However, each is significantly enriched for antisense sequences present in endo-siRNAs (Nam and Bartel 2012) and may function in a conserved silencing mechanism by acting as a template for the corresponding siRNA. The binding of FBF to ncRNA indicates that PUFs may target and control RNAs outside of the context of translational control. Of note, recent work in human cells demonstrates that a lincRNA can interact with PUM2 to modulate its RNA binding and target repression activities (Tichon et al. 2015).

To generate a large-scale view of relationships between proteins encoded by mRNAs in the FBF-regulated RNA network, we derived an interaction map of putative protein interactions from the STRING database (version 10.0, [Szklarczyk et al. 2015]). We considered only high-confidence interactions supported by experimental evidence, either with *C. elegans* proteins directly or with homologs. 347 of 1404 FBF targets had at least one interaction with another target (965 total interactions) and thereby formed a network of FBF target encoded protein interactions. Indeed, certain targets had many interactions within that network and were therefore “hubs,” with the top ten hubs presented in Figure 8A. Those top hubs include key mitotic and meiotic regulators (RAD-51, PCN-1, CDK-1, ATM-1, FZR-1, HSR-9, and MEL-26). Since FBF binds more than a thousand targets, repression of targets that act as hubs may be a mechanism to exert control over the entire network.

In summary, our combined FBF list revealed aspects of FBF target identity that were not previously known: that FBF directly binds to cell cycle regulators above any other biological process class, that FBF can bind ncRNAs, including several lincRNAs, and that FBF binds transcripts in vivo encoding itself and other *C. elegans* PUF proteins. We found other diverse targets from many key regulatory pathways (e.g., the MAP kinase and Wnt pathways), indicating that FBF is a multifunctional regulator and supporting a role for FBF as a regulator of GSC totipotency.

## DISCUSSION

We used iCLIP to determine the PUF binding landscapes for *C. elegans* FBF-1 and FBF-2 in intact animals and in their native context—namely GSCs. We first optimized the peak-calling method and then analyzed both data sets for key parameters. Our findings provide insights into the nature of sequences bound by these nearly identical PUF paralogs and the locations of their binding sites within their targets. It also defines a robust FBF-RNA regulatory network, biological functions of FBF targets, and conserved aspects of the network.

Our analysis reveals sites of FBF binding in germ cells on a genome-wide scale. First, FBF-1 and FBF-2 bind to targets not only via the established canonical motif UGUNNNAU, defined largely in vitro and in yeast (Bernstein et al. 2005; Opperman et al. 2005; Campbell et al. 2012), but also via previously unknown alternate sites. Alternate sites increase the repertoire of in vivo FBF binding elements to include half-mers, U- and G-enriched motifs, and a 7-mer enriched in peaks that lack an FBE. A similar 7-mer was previously found to bind in vitro with ∼20-fold lower affinity than the canonical site (Opperman et al. 2005). The greater number and diversity of FBF binding motifs identified in this work may enable fine-tuning of FBF-RNA interactions. Moreover, recent evidence from yeast indicates that PUF binding at short motif elements has small repressive effects (∼2%) on target mRNAs, suggesting subtle regulatory effects from binding, even at alternate motifs (Porter et al. 2015). Second, most FBF binding sites are in the 3′UTR and are enriched toward the terminal end of those UTRs. The bias toward the 3′ ends is consistent with the ability of PUF proteins to recruit a deadenylase to the end of target transcripts (Merritt et al. 2008; Weidmann et al. 2014). Although 3′ termini may be more accessible to FBF binding in vivo, major binding site peaks clearly occur across the whole transcript, implying that regions throughout the transcript can mediate PUF control. Recent studies of GLD-1, a conserved *C. elegans* RNA binding protein, demonstrated action via sites in both 5′ and 3′UTRs (Jungkamp et al. 2011), and regulatory elements in protein-coding regions have been found for miRNAs and the RNA binding proteins UPF1 and FMRP (Darnell et al. 2011; Hausser et al. 2013; Zund et al. 2013). Many FBF targets had primary and secondary peaks, with some containing over 15 peaks. Systematic manipulations of locations and numbers of binding sites are needed to assess their consequences for binding and biological control.

**FIGURE 9.**
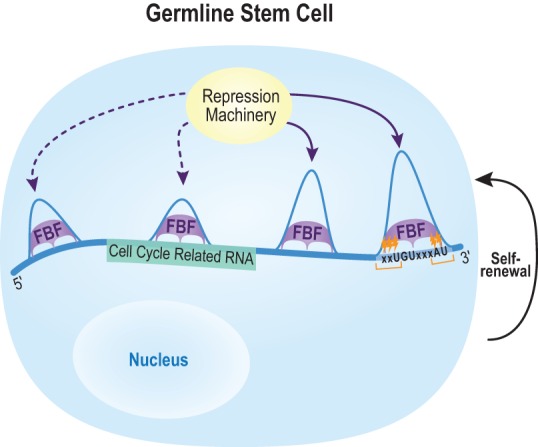
Model of in vivo FBF-mediated RNA regulation. FBF associates with RNA motifs to repress its targets (especially transcripts that encode cell cycle regulators as depicted here), and thereby promote GSC self-renewal. Our work expands the repertoire of FBF binding (shown with relative peak heights) to locations other than the 3′UTR, to lincRNAs, and to motifs other than the canonical FBE. Enriched crosslinking regions (orange marks) indicate regions of close protein–RNA interaction in vivo. Our model depicts multiple FBFs simultaneously bound on a single RNA, but a caveat from our data is that multiple peaks may represent a population of single binding events.

The RNA targets of FBF-1 and FBF-2 are strikingly similar and directly relevant to stem cell regulation. These paralogs bind the same target RNAs, bind the same locations within those RNAs, bind the same motifs and with similar frequency. In general, only minor differences in read enrichment were found between FBF-1 and FBF-2. These common binding landscapes reveal the molecular basis of the biological redundancy of these two PUF paralogs (Crittenden et al. 2002). The cell cycle emerged as the major biological process among FBF target mRNAs. Moreover, cell cycle regulators are enriched as conserved targets for both nematode and human PUFs (this work, Hafner et al. 2010), consistent with the idea that the ancient function of the PUF family is to regulate stem cell self-renewal (Wickens et al. 2002). Besides its expansive control over the cell cycle, FBF regulates a spectrum of previously unknown targets, including lincRNAs, the RNA degradation machinery, and components of miRNA biogenesis. In addition, our work more than doubles the number of germline sex determination RNAs under likely FBF regulation (from 10 to 22) and adds additional components to developmental signaling pathways whose control is likely central to stem cell regulation (Kimble and Seidel 2013; Schedl 2013). We suggest the model depicted in Figure 9 as a summary of how FBF controls GSCs: FBF binds cell cycle related RNAs primarily at the FBE in mRNA 3′UTRs, secondarily at other motifs in the 3′UTR, and more rarely at other positions in the RNA. These findings set the stage for an in depth biological analysis of post-transcriptional regulation in stem cells.

## MATERIALS AND METHODS

### Nematode strains used in this study

N2: wild-type *Caenorhabditis elegans*, Bristol strain
JK5181: *fbf-1(ok91) qSi232[3xflag::fbf-1] II*
JK5182: *fbf-2(q738) qSi75[3xflag::fbf-2] II*
JK5183: *fbf-1(ok91) fbf-2(q704) qSi232 II*
JK5032: *fbf-1(ok91) fbf-2(q704) qSi75 II*

### Generation of strains carrying epitope-tagged FBF-1 and FBF-2 transgenes

For 3xFLAG::FBF-1*,* the complete *fbf-1* genomic sequence (1.1 kb of the 5′ upstream region including the complete 5′UTR, all exons and introns, and 580 bp of the 3′ downstream region including the complete 3′UTR), plus an insert of 3xFLAG at the 5′ end of the coding sequence, was cloned into pCFJ151 (Frøkjær-Jensen et al. 2008) to create pJK1736. For 3xFLAG::FBF-2, the complete *fbf-2* genomic sequence (1.5 kb of the 5′ upstream region, including the complete 5′UTR, all exons and introns, and 1.1 kb of the 3′ downstream region, including the complete 3′UTR) plus an insert of 3xFLAG at the 5′ end of the coding sequence was cloned into pCFJ151 to create pJK1726. pJK1736 and pJK1726 were used to generate *qSi232* and *qSi75* transgenes, respectively. Transgenes were inserted into the *ttTi5605* site on *LGII* of strain EG6699 using the *Mos1*-mediated single copy insertion (MosSCI) method (Frøkjær-Jensen et al. 2008). *qSi232* (3xFLAG::FBF-1) and *qSi75* (3xFLAG::FBF-2) were introduced into single or double mutant backgrounds to generate strains JK5181, JK5182, JK5183, and JK5032 by standard genetic crosses. The presence of each mutant and homozygosity were verified after multiple generations by PCR for deletion mutants and/or Sanger sequencing.

### iCLIP

iCLIP was carried out as described (Huppertz et al. 2014) with modifications for worm growth and lysis described here. Single-end sequencing was performed on an Illumina HiSeq 2000.

#### Nematode culture for iCLIP

*Caenorhabditis elegans* strains N2, JK5181, or JK5182 were cultivated at 20°C and grown to early adulthood (i.e., 24 h after L4) in all iCLIP experiments (Brenner 1974). Wild-type N2 (Bristol strain) served as a negative control for tagged FBF transgenes. Developmental stage was evaluated by examining animals with a Zeiss Axio Imager D.1 compound microscope at 10× and 40× magnification for body size and stage-specific markers (e.g., vulva formation). Animals were kept on standard NGM plates and fed *E. coli* OP50 as previously described (Stiernagle 2006). For growth, age-synchronized first stage larvae (L1) were obtained by standard methods. Briefly, gravid unlabeled adults were treated with 2:1 bleach:5 N NaOH to isolate embryos (Lewis and Fleming 1995). The embryos were incubated in M9 buffer (per 1 L of buffer: 6 g Na_2_HPO_4_, 3 g KH_2_PO_4_, 5 g NaCl, 1 mL of 1 M MgSO_4_) and then resuspended in M9 without food in a ventilated Erlenmeyer flask at 20°C for 18 h, shaking at 170 RPM to obtain synchronized L1 larvae. The unlabeled L1s were pelleted at 2500 RCF for 2 min, washed twice with 15 mL of M9, resuspended in 10 mL of M9, and then distributed to 10 cm NGM plates pre-equilibrated to 20°C. Plates (10 cm) were seeded with 1.5 mL of 30× *E. coli* OP50. L1s were added 24 h after seeding (∼6000 per plate, as estimated via extrapolation from an aliquot of the washed, synchronized L1 culture). We grew pellets of ∼50,000 adult worms.

#### UV crosslinking

Upon reaching the adult stage, live worms were quickly rinsed from plates into a 15 mL falcon tube with cold M9, washed once with cold M9, pelleted at 201 RCF in 4°C M9 and then transferred by Pasteur pipette to cold, unseeded 10 cm NGM plates with the minimum amount of liquid. Animals were irradiated three times sequentially at 254 nm with 400 mJ/cm^2^ in a Spectrolinker XL-1000 UV Crosslinker with the plate cover removed. The total crosslinking time was ∼5 min. Worms were rinsed from the plates with cold M9, pelleted at 201 RCF for 1 min, transferred to a 2 mL tube, and snap frozen in liquid nitrogen. Pellets were stored at −80°C.

#### Lysis and partial RNA digestion

*Caenorhabditis elegans* pellets were thawed by adding 800 µL of ice-cold iCLIP lysis buffer (Huppertz et al. 2014) and mutated for 20 min at 4°C. The thawed pellets were centrifuged at 1000 RCF and 4°C for 1 min and washed three times with 800 µL of ice-cold lysis buffer. One mL of lysis buffer was added to the pellet along with a 5-mm stainless steel ball (Retsch). Lysis was performed in the cold room using a Retsch 400 MM mill mixer. Lysis was completed after three 10-min cycles at a setting of 30 Hz, with 4-min freeze–thaws after the first and second cycles. Freeze–thaws were performed by immersion in liquid nitrogen for 1 min, then returning to liquid state by immersion in room temperature water for 4 min. We prevented cracking of the tube lid during such aggressive bead lysis by using strong RNase-free tubes that were not autoclaved (USA Scientific Cat. No. 1620-2700) and by placing a small square of reinforced tape, such as Gorilla Tape, over the tube lid just prior to bead lysis. This method yielded complete tissue lysis as confirmed by observing a small aliquot of final lysate at 40× magnification. Partial RNA digestion was performed as previously described (Huppertz et al. 2014) with a 1:250 RNase I dilution. Lysate was then cleared by centrifugation for 15 min at 16,100 RCF and 4°C. Protein concentration of the cleared lysate was determined with the Direct Detect spectrometer (Millipore). Our pellets containing ∼50,000 worms yielded ∼10 mg/mL of total protein, and we used 10 mg total protein per biological replicate in iCLIP experiments.

### Immunocytochemistry

Antibody staining of dissected gonads was carried out essentially as described (Lee et al. 2006). Briefly, dissected gonads were fixed and permeabilized in 1 mL of pre-chilled 100% methanol at −20°C for 10 min. Gonads were then immediately pelleted by centrifugation at 376 RCF for 2 min and treated with 1 mL of pre-chilled 100% acetone at −20°C for 10 min. Samples were washed twice with 1 mL of PBST 0.01% (1× concentrated PBS plus 0.01% vol/vol Triton X-100) and blocked in 1 mL of PBST 0.1% (1× PBS plus 0.1% vol/vol Triton X-100) plus 3% wt/vol BSA for 30 min at room temperature. Samples were then incubated at 4°C overnight with monoclonal anti-FLAG M2 antibody produced in mouse (Sigma) at a concentration of 1:1000 in 100 µL of PBST (PBS plus 0.1% Triton X-100 plus 3% BSA). Samples were washed three times for 5 min with 1 mL of PBST (0.1% Triton X-100) and then incubated for 2 h at room temperature with Alexa 488 conjugated secondary antibody (Jackson ImmunoResearch) at a concentration of 1:500 in 100 µL of PBST (0.1% Triton X-100 plus 3% BSA). 4′,6-diamidino-2-phenylindole (DAPI) (0.5 ng/μL) was included to visualize DNA. Confocal images were taken using a Leica TCS SP8. Antibody staining of dissected gonads for phospho-histone H3 (a marker of actively dividing cells) was carried out as described (Seidel and Kimble 2015).

### RNAi experiments

RNAi feeding experiments were carried out following established protocols (Kershner et al. 2014). For multiple gene knockdowns, HT115 bacteria containing *lst-1* or *sygl-1* RNAi vectors were grown separately in overnight cultures and then seeded to RNAi plates in equal volumes. As a control, we used empty RNAi vector (pL4440).

### Mass spectrometry

Worm lysis and 3xFLAG::FBF-1 or 3xFLAG::FBF-2 immunoprecipitation (including the stringent bead washes) was carried out as for iCLIP. Captured FBF-1 or FBF-2 was eluted from the Protein G Dynabeads with 50 µL of 0.1 M glycine (pH 3.0) buffer (shaking at 1100 rpm in a Thermomixer R at room temperature for 6 min) and then immediately neutralized with 5 µL of Tris–HCl pH 7.5. Two biological replicates were performed for each FBF and two negative control N2 immunoprecipitations were carried out in parallel. Eluates were pooled before enzymatic digestion. Prior to mass spectrometry, we verified the presence of 3xFLAG::FBF-1 and 3xFLAG::FBF-2 in the desired eluates by Western blotting. Importantly, we were unable to obtain enough protein to visualize by colloidal Coomassie staining or silver staining of protein gels, consistent with the relatively low abundance of FBF-1 and FBF-2 in the animal. However, we did not find the failure to visualize the proteins by gel staining to be a limitation for accurate and specific mass spectrometry.

#### Enzymatic digestion

Roughly 5 µg of total protein was TCA/acetone precipitated (10% TCA, 28% acetone final), then pellets resolubilized, and denatured in 7.5 μL of 8 M urea/50 mM NH_4_HCO_3_ (pH 8.5)/1 mM Tris–HCl for 5 min. Solubilized pellets were diluted to 30 μL for reduction with 1.25 μL of 25 mM DTT, 2.5 μL of MeOH, 18.75 μL of 25 mM NH_4_HCO_3_ (pH 8.5), incubated for 15 min at 50°C, cooled on ice to room temperature, and then 1.5 μL of 55 mM IAA was added for alkylation. Samples were incubated in darkness at room temperature for 15 min. The alkylation reaction was quenched by adding 4 μL of 25 mM DTT. Subsequently, 0.75 μL of trypsin/LysC solution (100 ng/μL trypsin/LysC Mix from Promega Corp. in 25 mM NH_4_HCO_3_) and 13.5 μL of 25 mM NH_4_HCO_3_ (pH 8.5) were added to a 50 µL final volume. Digestion was conducted for 2 h at 42°C, and then an additional 0.4 µL of trypsin/LysC solution added (final enzyme:substrate ∼1:40 with an estimated ∼5 µg substrate) and digestion proceeded overnight at 37°C. The reaction was terminated by acidification with 2.5% TFA (trifluoroacetic acid) to a 0.3% final concentration.

#### NanoLC-MS/MS

The digest was cleaned using OMIX C18 SPE cartridges (Agilent) per the manufacturer's protocol and eluted in 20 µL of 60/40/0.1% ACN/H_2_O/TFA, dried to completion in a Speed-vac and finally reconstituted in 15 µL of 0.1% formic acid. Peptides were analyzed by nanoLC-MS/MS using the Agilent 1100 nanoflow system (Agilent) connected to a hybrid linear ion trap-orbitrap mass spectrometer (LTQ-Orbitrap Elite, Thermo Fisher Scientific) equipped with an EASY-Spray electrospray source. Chromatography of peptides prior to mass spectral analysis was accomplished using a capillary emitter column (PepMap C18, 3 µM, 100 Å, 150 × 0.075 mm, Thermo Fisher Scientific) onto which 3 µL of extracted peptides was automatically loaded. To load the peptides onto the column, the NanoHPLC system delivered solvent A (0.1% (v/v) formic acid) and solvent B (99.9% (v/v) acetonitrile, 0.1% (v/v) formic acid) at 0.60 µL/min over a 45 min period. To elute the peptides from the column directly into the nano-electrospray, the system delivered solvent at 0.3 µL/min with a gradual gradient from 0% (v/v) B to 35% (v/v) B over 140 min and concluded with a 10 min fast gradient from 35% (v/v) B to 60% (v/v) B. Then, a 7 min flash-out from 60% to 100% (v/v) B took place. As peptides eluted from the HPLC-column/electrospray source, MS scans were acquired in the Orbitrap with a resolution of 120,000, followed by MS2 fragmentation of the 20 most intense peptides detected in the MS1 scan from 300 to 2000 *m*/*z*; redundancy was limited by dynamic exclusion. Raw MS/MS data were converted to mgf file format using MSConvert (ProteoWizard: Open Source Software for Rapid Proteomics Tools Development). Resulting mgf files were used to search against the *C. elegans* database with decoy reverse entries (51,351 total entries) using an in-house *Mascot* search engine 2.2.07 (Matrix Science) with variable methionine oxidation, asparagine/glutamine deamidation and serine/threonine phosphorylation. Peptide mass tolerance was set at 15 ppm and fragment mass at 0.6 Da. Protein annotations and significance of identification were done with the help of Scaffold software (version 4.3.2, Proteome Software Inc.). Protein identifications were accepted if they could be established at >95.0% probability, within 1% false discovery rate, and contained at least two identified peptides. Protein probabilities were assigned by the Protein Prophet algorithm (Nesvizhskii et al. 2003). Proteins that contained similar peptides and could not be differentiated based on MS–MS analysis alone were grouped to satisfy the principles of parsimony. Scaffold's spectral counting strategy was employed to compare relative protein abundances between the samples.

### Read mapping

Adapters and duplicate reads were removed using Python scripts and reads mapped to the WS235 genome using bowtie2 (using the parameter –local) (Langmead and Salzberg 2012). Mapped reads with a MAPQ quality score below 20 were removed. Mapped reads from replicate N2 data sets were combined to form single negative IP data sets.

### iCLIP peak assignment and motif analysis

A previously published algorithm was used to identify putative peaks (Du et al. 2006). Overlapping peaks were merged and the borders of the peak were placed where read density fell to 20% of the peak height. Reads were placed in 50-nt bins according to their 5′ position. Control RNA-seq data were obtained from MODENCODE (accession: modEncode_4594), representing total RNA from animals at a similar developmental time point as in this study. Initially, signal from RNA-seq and negative control iCLIP in the local 500-bp genomic region were modeled as a Gaussian or a negative binomial to calculate the *P*-value for obtaining a bin with as many reads as the maximum iCLIP bin. As an alternative, a Gaussian, Poisson, or negative binomial was also modeled for all bins in the mature RNA of the target gene to calculate a *P*-value for obtaining a given peak height in the gene of interest. Given the added computational time of negative binomial modeling and its similar performance to the Gaussian modeling, we opted to use the Gaussian modeling for further analyses. Signal from FBF iCLIP itself was modeled as a Poisson in all cases. A Bonferroni correction for each peak *P*-value was applied by multiplying the peak *P*-value by the number of bins used for modeling. A Benjamini-Hochberg correction was applied to the *P*-values in the set of all potential peaks at an FDR cutoff of 1%. To generate FBF-1 and FBF-2 target lists, iCLIP replicates were combined by requiring the same peak be called in all three biological replicates. We then applied an additional secondary filter, comparing the reads overlapping the peak region in the FBF iCLIP data with the negative control iCLIP in the same region, and requiring at least a fivefold enrichment. The negative control was assigned one read if it contained no reads. We also considered the use of the RNA-seq data for the secondary filter, but it did not yield an improvement in the peak calling. A statistically strong list of “FBF targets” was generated in the same way except that the same peaks were required in five out of six FBF-1 plus FBF-2 replicates. MEME was used to search for motifs of length 4–9 (Bailey et al. 2009). For MEME searches, the algorithm of Du et al. (2006) was applied to “un-merge” merged peak regions and to count all distinct interactions. MEME was queried with 71-nt sequences centered around each peak maxima. The filtering of individual FBF-1 and FBF-2 targets was by a normalized reads-in-peak ratio of 3 for merged peak regions. Because the merged peak regions are larger than those of individual replicates, and are more likely to pass filter than the individual replicate peak regions, we lowered the filter for individual replicates from 3 to 2.5 to produce the Venn diagrams of biological replicates targets in Figure 3B.

### Crosslinking-induced mutation site (CIMS) and crosslinking-induced truncation site (CITS) analyses

CIMS and CITS were performed as previously described (Weyn-Vanhentenryck et al. 2014), with additional Python code. Reads were mapped, using NovoAlign, to WS235. Reads were collapsed according to the random barcode by EM algorithm. Consistent with other CLIP studies, we found that only deletions and truncations identified realistic binding sites, so we restricted our analysis to these mutations.

### Gene Ontology (GO) and STRING analysis

The Database for Annotation, Visualization, and Integrated Discovery (DAVID, v. 6.7) was used to identify enriched GO terms within the FBF target list (Huang da et al. 2009a,b). The Bonferroni test for multiple hypothesis testing was applied to *P*-values. To generate a large-scale view of relationships within the FBF-regulated RNA network, we derived an interaction map using the STRING database (version 10.0 [Szklarczyk et al. 2015]). We set the organism as *C. elegans* with a “high” confidence setting and considered interactions only if they were supported by experimental evidence, either with *C. elegans* proteins directly or with homologs.

## DATA DEPOSITION

Raw sequencing data can be found in the Gene Expression Omnibus database with accession number GSE76136. Custom code used for peak assignment and analysis can be found at github.com/dfporter. Worm strains used in this study are available upon request.

## SUPPLEMENTAL MATERIAL

Supplemental material is available for this article.

## Supplementary Material

Supplemental Material

## References

[PRASADRNA055871C1] Ahringer J, Kimble J. 1991 Control of the sperm-oocyte switch in *Caenorhabditis elegans* hermaphrodites by the *fem-*3 3′ untranslated region. Nature 349: 346–348. 1702880 10.1038/349346a0

[PRASADRNA055871C2] Anko ML, Muller-McNicoll M, Brandl H, Curk T, Gorup C, Henry I, Ule J, Neugebauer KM. 2012 The RNA-binding landscapes of two SR proteins reveal unique functions and binding to diverse RNA classes. Genome Biol 13: R17. 22436691 10.1186/gb-2012-13-3-r17 PMC3439968

[PRASADRNA055871C3] Bailey TL, Boden M, Buske FA, Frith M, Grant CE, Clementi L, Ren J, Li WW, Noble WS. 2009 MEME SUITE: tools for motif discovery and searching. Nucleic Acids Res 37: W202–W208. 19458158 10.1093/nar/gkp335 PMC2703892

[PRASADRNA055871C4] Bernstein D, Hook B, Hajarnavis A, Opperman L, Wickens M. 2005 Binding specificity and mRNA targets of a *C. elegans* PUF protein, FBF-1. RNA 11: 447–458. 15769874 10.1261/rna.7255805 PMC1370734

[PRASADRNA055871C5] Brenner S. 1974 The genetics of *Caenorhabditis elegans*. Genetics 77: 71–94. 4366476 10.1093/genetics/77.1.71 PMC1213120

[PRASADRNA055871C6] Buratti E, Baralle FE. 2011 TDP-43: new aspects of autoregulation mechanisms in RNA binding proteins and their connection with human disease. FEBS J 278: 3530–3538. 21777388 10.1111/j.1742-4658.2011.08257.x

[PRASADRNA055871C7] Campbell ZT, Bhimsaria D, Valley CT, Rodriguez-Martinez JA, Menichelli E, Williamson JR, Ansari AZ, Wickens M. 2012 Cooperativity in RNA-protein interactions: global analysis of RNA binding specificity. Cell Rep 1: 570–581. 22708079 10.1016/j.celrep.2012.04.003 PMC3375920

[PRASADRNA055871C9] Chen P, Ellis RE. 2000 TRA-1A regulates transcription of *fog-3*, which controls germ cell fate in *C. elegans*. Development 127: 3119–3129. 10862749 10.1242/dev.127.14.3119

[PRASADRNA055871C8] Chen D, Zheng W, Lin A, Uyhazi K, Zhao H, Lin H. 2012 Pumilio 1 suppresses multiple activators of p53 to safeguard spermatogenesis. Curr Biol 22: 420–425. 22342750 10.1016/j.cub.2012.01.039 PMC3449084

[PRASADRNA055871C10] Chi SW, Zang JB, Mele A, Darnell RB. 2009 Argonaute HITS-CLIP decodes microRNA-mRNA interaction maps. Nature 460: 479–486. 19536157 10.1038/nature08170 PMC2733940

[PRASADRNA055871C11] Crittenden SL, Bernstein DS, Bachorik JL, Thompson BE, Gallegos M, Petcherski AG, Moulder G, Barstead R, Wickens M, Kimble J. 2002 A conserved RNA-binding protein controls germline stem cells in *Caenorhabditis elegans*. Nature 417: 660–663. 12050669 10.1038/nature754

[PRASADRNA055871C12] Darnell RB. 2010 HITS-CLIP: panoramic views of protein-RNA regulation in living cells. Wiley Interdiscip Rev RNA 1: 266–286. 21935890 10.1002/wrna.31 PMC3222227

[PRASADRNA055871C13] Darnell JC, Van Driesche SJ, Zhang C, Hung KY, Mele A, Fraser CE, Stone EF, Chen C, Fak JJ, Chi SW, 2011 FMRP stalls ribosomal translocation on mRNAs linked to synaptic function and autism. Cell 146: 247–261. 21784246 10.1016/j.cell.2011.06.013 PMC3232425

[PRASADRNA055871C14] Dredge BK, Stefani G, Engelhard CC, Darnell RB. 2005 Nova autoregulation reveals dual functions in neuronal splicing. EMBO J 24: 1608–1620. 15933722 10.1038/sj.emboj.7600630 PMC1142566

[PRASADRNA055871C15] Du P, Kibbe WA, Lin SM. 2006 Improved peak detection in mass spectrum by incorporating continuous wavelet transform-based pattern matching. Bioinformatics 22: 2059–2065. 16820428 10.1093/bioinformatics/btl355

[PRASADRNA055871C16] Friedersdorf MB, Keene JD. 2014 Advancing the functional utility of PAR-CLIP by quantifying background binding to mRNAs and lncRNAs. Genome Biol 15: R2. 24393468 10.1186/gb-2014-15-1-r2 PMC4053780

[PRASADRNA055871C17] Friend K, Campbell ZT, Cooke A, Kroll-Conner P, Wickens MP, Kimble J. 2012 A conserved PUF-Ago-eEF1A complex attenuates translation elongation. Nat Struct Mol Biol 19: 176–183. 22231398 10.1038/nsmb.2214 PMC3293257

[PRASADRNA055871C18] Frøkjær-Jensen C, Davis MW, Hopkins CE, Newman BJ, Thummel JM, Olesen SP, Grunnet M, Jorgensen EM. 2008 Single-copy insertion of transgenes in *Caenorhabditis elegans*. Nat Genet 40: 1375–1383. 18953339 10.1038/ng.248 PMC2749959

[PRASADRNA055871C19] Galgano A, Forrer M, Jaskiewicz L, Kanitz A, Zavolan M, Gerber AP. 2008 Comparative analysis of mRNA targets for human PUF-family proteins suggests extensive interaction with the miRNA regulatory system. PLoS One 3: e3164. 18776931 10.1371/journal.pone.0003164 PMC2522278

[PRASADRNA055871C20] Gerber AP, Herschlag D, Brown PO. 2004 Extensive association of functionally and cytotopically related mRNAs with Puf family RNA-binding proteins in yeast. PLoS Biol 2: E79. 15024427 10.1371/journal.pbio.0020079 PMC368173

[PRASADRNA055871C21] Gerber AP, Luschnig S, Krasnow MA, Brown PO, Herschlag D. 2006 Genome-wide identification of mRNAs associated with the translational regulator PUMILIO in *Drosophila melanogaster*. Proc Natl Acad Sci 103: 4487–4492. 16537387 10.1073/pnas.0509260103 PMC1400586

[PRASADRNA055871C22] Goldstrohm AC, Wickens M. 2008 Multifunctional deadenylase complexes diversify mRNA control. Nat Rev Mol Cell Biol 9: 337–344. 18334997 10.1038/nrm2370

[PRASADRNA055871C23] Goldstrohm AC, Hook BA, Seay DJ, Wickens M. 2006 PUF proteins bind Pop2p to regulate messenger RNAs. Nat Struct Mol Biol 13: 533–539. 16715093 10.1038/nsmb1100

[PRASADRNA055871C24] Hafner M, Landthaler M, Burger L, Khorshid M, Hausser J, Berninger P, Rothballer A, Ascano M Jr, Jungkamp AC, Munschauer M, 2010 Transcriptome-wide identification of RNA-binding protein and microRNA target sites by PAR-CLIP. Cell 141: 129–141. 20371350 10.1016/j.cell.2010.03.009 PMC2861495

[PRASADRNA055871C25] Hausser J, Syed AP, Bilen B, Zavolan M. 2013 Analysis of CDS-located miRNA target sites suggests that they can effectively inhibit translation. Genome Res 23: 604–615. 23335364 10.1101/gr.139758.112 PMC3613578

[PRASADRNA055871C26] Henderson MA, Cronland E, Dunkelbarger S, Contreras V, Strome S, Keiper BD. 2009 A germline-specific isoform of eIF4E (IFE-1) is required for efficient translation of stored mRNAs and maturation of both oocytes and sperm. J Cell Sci 122: 1529–1539. 19383718 10.1242/jcs.046771 PMC2680099

[PRASADRNA055871C27] Hogan GJ, Brown PO, Herschlag D. 2015 Evolutionary conservation and diversification of Puf RNA binding proteins and their mRNA targets. PLoS Biol 13: e1002307. 26587879 10.1371/journal.pbio.1002307 PMC4654594

[PRASADRNA055871C28] Huang da W, Sherman BT, Lempicki RA. 2009a Bioinformatics enrichment tools: paths toward the comprehensive functional analysis of large gene lists. Nucleic Acids Res 37: 1–13. 19033363 10.1093/nar/gkn923 PMC2615629

[PRASADRNA055871C29] Huang da W, Sherman BT, Lempicki RA. 2009b Systematic and integrative analysis of large gene lists using DAVID bioinformatics resources. Nat Protoc 4: 44–57. 19131956 10.1038/nprot.2008.211

[PRASADRNA055871C30] Huppertz I, Attig J, D'Ambrogio A, Easton LE, Sibley CR, Sugimoto Y, Tajnik M, Konig J, Ule J. 2014 iCLIP: protein-RNA interactions at nucleotide resolution. Methods 65: 274–287. 24184352 10.1016/j.ymeth.2013.10.011 PMC3988997

[PRASADRNA055871C31] Jangi M, Boutz PL, Paul P, Sharp PA. 2014 Rbfox2 controls autoregulation in RNA-binding protein networks. Genes Dev 28: 637–651. 24637117 10.1101/gad.235770.113 PMC3967051

[PRASADRNA055871C32] Jungkamp AC, Stoeckius M, Mecenas D, Grun D, Mastrobuoni G, Kempa S, Rajewsky N. 2011 In vivo and transcriptome-wide identification of RNA binding protein target sites. Mol Cell 44: 828–840. 22152485 10.1016/j.molcel.2011.11.009 PMC3253457

[PRASADRNA055871C33] Kadyrova LY, Habara Y, Lee TH, Wharton RP. 2007 Translational control of maternal *Cyclin B* mRNA by Nanos in the *Drosophila* germline. Development 134: 1519–1527. 17360772 10.1242/dev.002212

[PRASADRNA055871C34] Kaye JA, Rose NC, Goldsworthy B, Goga A, L'Etoile ND. 2009 A 3′UTR pumilio-binding element directs translational activation in olfactory sensory neurons. Neuron 61: 57–70. 19146813 10.1016/j.neuron.2008.11.012 PMC4274156

[PRASADRNA055871C35] Keene JD. 2007 RNA regulons: coordination of post-transcriptional events. Nat Rev Genet 8: 533–543. 17572691 10.1038/nrg2111

[PRASADRNA055871C36] Keene JD, Tenenbaum SA. 2002 Eukaryotic mRNPs may represent posttranscriptional operons. Mol Cell 9: 1161–1167. 12086614 10.1016/s1097-2765(02)00559-2

[PRASADRNA055871C37] Kershaw CJ, Costello JL, Talavera D, Rowe W, Castelli LM, Sims PF, Grant CM, Ashe MP, Hubbard SJ, Pavitt GD. 2015 Integrated multi-omics analyses reveal the pleiotropic nature of the control of gene expression by Puf3p. Sci Rep 5: 15518. 26493364 10.1038/srep15518 PMC4616039

[PRASADRNA055871C38] Kershner AM, Kimble J. 2010 Genome-wide analysis of mRNA targets for *Caenorhabditis elegans* FBF, a conserved stem cell regulator. Proc Natl Acad Sci 107: 3936–3941. 20142496 10.1073/pnas.1000495107 PMC2840422

[PRASADRNA055871C39] Kershner AM, Shin H, Hansen TJ, Kimble J. 2014 Discovery of two GLP-1/Notch target genes that account for the role of GLP-1/Notch signaling in stem cell maintenance. Proc Natl Acad Sci 111: 3739–3744. 24567412 10.1073/pnas.1401861111 PMC3956202

[PRASADRNA055871C40] Kimble J, Seidel H. 2013 *C. elegans* germline stem cells and their niche. In StemBook. Harvard Stem Cell Institute, Cambridge, MA. 24354021

[PRASADRNA055871C41] Kishore S, Jaskiewicz L, Burger L, Hausser J, Khorshid M, Zavolan M. 2011 A quantitative analysis of CLIP methods for identifying binding sites of RNA-binding proteins. Nat Methods 8: 559–564. 21572407 10.1038/nmeth.1608

[PRASADRNA055871C42] Konig J, Zarnack K, Rot G, Curk T, Kayikci M, Zupan B, Turner DJ, Luscombe NM, Ule J. 2010 iCLIP reveals the function of hnRNP particles in splicing at individual nucleotide resolution. Nat Struct Mol Biol 17: 909–915. 20601959 10.1038/nsmb.1838 PMC3000544

[PRASADRNA055871C43] Lamont LB, Crittenden SL, Bernstein D, Wickens M, Kimble J. 2004 FBF-1 and FBF-2 regulate the size of the mitotic region in the *C. elegans* germline. Dev Cell 7: 697–707. 15525531 10.1016/j.devcel.2004.09.013

[PRASADRNA055871C44] Lander AD, Kimble J, Clevers H, Fuchs E, Montarras D, Buckingham M, Calof AL, Trumpp A, Oskarsson T. 2012 What does the concept of the stem cell niche really mean today? BMC Biol 10: 19. 22405133 10.1186/1741-7007-10-19 PMC3298504

[PRASADRNA055871C45] Langmead B, Salzberg SL. 2012 Fast gapped-read alignment with Bowtie 2. Nat Methods 9: 357–359. 22388286 10.1038/nmeth.1923 PMC3322381

[PRASADRNA055871C46] Lapointe CP, Wilinski D, Saunders HA, Wickens M. 2015 Protein–RNA networks revealed through covalent RNA marks. Nat Methods 12: 1163–1170. 26524240 10.1038/nmeth.3651 PMC4707952

[PRASADRNA055871C47] Lee MH, Hook B, Lamont LB, Wickens M, Kimble J. 2006 LIP-1 phosphatase controls the extent of germline proliferation in *Caenorhabditis elegans*. EMBO J 25: 88–96. 16319922 10.1038/sj.emboj.7600901 PMC1351240

[PRASADRNA055871C48] Lewis JA, Fleming JT. 1995 Basic culture methods. Methods Cell Biol 48: 3–29. 8531730

[PRASADRNA055871C49] Licatalosi DD, Mele A, Fak JJ, Ule J, Kayikci M, Chi SW, Clark TA, Schweitzer AC, Blume JE, Wang X, 2008 HITS-CLIP yields genome-wide insights into brain alternative RNA processing. Nature 456: 464–469. 18978773 10.1038/nature07488 PMC2597294

[PRASADRNA055871C50] Lin H, Spradling AC. 1997 A novel group of *pumilio* mutations affects the asymmetric division of germline stem cells in the *Drosophila* ovary. Development 124: 2463–2476. 9199372 10.1242/dev.124.12.2463

[PRASADRNA055871C51] Liu Q, Zhong X, Madison BB, Rustgi AK, Shyr Y. 2015 Assessing computational steps for CLIP-Seq data analysis. Biomed Res Int 2015: 196082. 26539468 10.1155/2015/196082 PMC4619761

[PRASADRNA055871C52] Luitjens C, Gallegos M, Kraemer B, Kimble J, Wickens M. 2000 CPEB proteins control two key steps in spermatogenesis in *C. elegans*. Genes Dev 14: 2596–2609. 11040214 10.1101/gad.831700 PMC316992

[PRASADRNA055871C53] Merritt C, Seydoux G. 2010 The Puf RNA-binding proteins FBF-1 and FBF-2 inhibit the expression of synaptonemal complex proteins in germline stem cells. Development 137: 1787–1798. 20431119 10.1242/dev.050799 PMC2867315

[PRASADRNA055871C54] Merritt C, Rasoloson D, Ko D, Seydoux G. 2008 3′ UTRs are the primary regulators of gene expression in the *C. elegans* germline. Curr Biol 18: 1476–1482. 18818082 10.1016/j.cub.2008.08.013 PMC2585380

[PRASADRNA055871C55] Mili S, Steitz JA. 2004 Evidence for reassociation of RNA-binding proteins after cell lysis: implications for the interpretation of immunoprecipitation analyses. RNA 10: 1692–1694. 15388877 10.1261/rna.7151404 PMC1370654

[PRASADRNA055871C56] Moore MJ, Silver PA. 2008 Global analysis of mRNA splicing. RNA 14: 197–203. 18083834 10.1261/rna.868008 PMC2212243

[PRASADRNA055871C57] Moore MJ, Zhang C, Gantman EC, Mele A, Darnell JC, Darnell RB. 2014 Mapping Argonaute and conventional RNA-binding protein interactions with RNA at single-nucleotide resolution using HITS-CLIP and CIMS analysis. Nat Protoc 9: 263–293. 24407355 10.1038/nprot.2014.012 PMC4156013

[PRASADRNA055871C58] Morris AR, Mukherjee N, Keene JD. 2008 Ribonomic analysis of human Pum1 reveals *cis-trans* conservation across species despite evolution of diverse mRNA target sets. Mol Cell Biol 28: 4093–4103. 18411299 10.1128/MCB.00155-08 PMC2423135

[PRASADRNA055871C59] Nam JW, Bartel DP. 2012 Long noncoding RNAs in *C. elegans*. Genome Res 22: 2529–2540. 22707570 10.1101/gr.140475.112 PMC3514682

[PRASADRNA055871C60] Nesvizhskii AI, Keller A, Kolker E, Aebersold R. 2003 A statistical model for identifying proteins by tandem mass spectrometry. Anal Chem 75: 4646–4658. 14632076 10.1021/ac0341261

[PRASADRNA055871C61] Nousch M, Techritz N, Hampel D, Millonigg S, Eckmann CR. 2013 The Ccr4-Not deadenylase complex constitutes the main poly(A) removal activity in *C. elegans*. J Cell Sci 126: 4274–4285. 23843623 10.1242/jcs.132936

[PRASADRNA055871C62] Opperman L, Hook B, DeFino M, Bernstein DS, Wickens M. 2005 A single spacer nucleotide determines the specificities of two mRNA regulatory proteins. Nat Struct Mol Biol 12: 945–951. 16244662 10.1038/nsmb1010

[PRASADRNA055871C63] Ortiz MA, Noble D, Sorokin EP, Kimble J. 2014 A new dataset of spermatogenic vs. oogenic transcriptomes in the nematode *Caenorhabditis elegans*. G3 (Bethesda) 4: 1765–1772. 25060624 10.1534/g3.114.012351 PMC4169169

[PRASADRNA055871C64] Porter DF, Koh YY, VanVeller B, Raines RT, Wickens M. 2015 Target selection by natural and redesigned PUF proteins. Proc Natl Acad Sci 112: 15868–15873. 26668354 10.1073/pnas.1508501112 PMC4703012

[PRASADRNA055871C65] Qiu C, Kershner A, Wang Y, Holley CP, Wilinski D, Keles S, Kimble J, Wickens M, Hall TM. 2012 Divergence of Pumilio/*fem-3* mRNA binding factor (PUF) protein specificity through variations in an RNA-binding pocket. J Biol Chem 287: 6949–6957. 22205700 10.1074/jbc.M111.326264 PMC3307254

[PRASADRNA055871C66] Quenault T, Lithgow T, Traven A. 2011 PUF proteins: repression, activation and mRNA localization. Trends Cell Biol 21: 104–112. 21115348 10.1016/j.tcb.2010.09.013

[PRASADRNA055871C67] Salvetti A, Rossi L, Lena A, Batistoni R, Deri P, Rainaldi G, Locci MT, Evangelista M, Gremigni V. 2005 *DjPum*, a homologue of *Drosophila Pumilio*, is essential to planarian stem cell maintenance. Development 132: 1863–1874. 15772127 10.1242/dev.01785

[PRASADRNA055871C68] Schedl T. 2013 Germ cell development in C. elegans. Springer, New York.

[PRASADRNA055871C69] Seidel HS, Kimble J. 2015 Cell-cycle quiescence maintains *Caenorhabditis elegans* germline stem cells independent of GLP-1/Notch. Elife 4: e10832. 26551561 10.7554/eLife.10832 PMC4718729

[PRASADRNA055871C70] Shaye DD, Greenwald I. 2011 OrthoList: a compendium of *C. elegans* genes with human orthologs. PLoS One 6: e20085. 21647448 10.1371/journal.pone.0020085 PMC3102077

[PRASADRNA055871C71] Spassov DS. 2004 “The role of Pumilio genes in maintenance and self-renewal of hematopoietic stem cells and progenitors.” PhD Thesis, University of Miami.

[PRASADRNA055871C72] Spradling A, Drummond-Barbosa D, Kai T. 2001 Stem cells find their niche. Nature 414: 98–104. 11689954 10.1038/35102160

[PRASADRNA055871C73] Stiernagle T. 2006 Maintenance of *C. elegans*. In WormBook (ed. The *C. elegans* Research Community), pp. 1–11. http://www.wormbook.org. 10.1895/wormbook.1.101.1 PMC4781397 18050451

[PRASADRNA055871C74] Sugimoto Y, Konig J, Hussain S, Zupan B, Curk T, Frye M, Ule J. 2012 Analysis of CLIP and iCLIP methods for nucleotide-resolution studies of protein-RNA interactions. Genome Biol 13: R67. 22863408 10.1186/gb-2012-13-8-r67 PMC4053741

[PRASADRNA055871C75] Suh N, Crittenden SL, Goldstrohm A, Hook B, Thompson B, Wickens M, Kimble J. 2009 FBF and its dual control of *gld-1* expression in the *Caenorhabditis elegans* germline. Genetics 181: 1249–1260. 19221201 10.1534/genetics.108.099440 PMC2666496

[PRASADRNA055871C76] Szklarczyk D, Franceschini A, Wyder S, Forslund K, Heller D, Huerta-Cepas J, Simonovic M, Roth A, Santos A, Tsafou KP, 2015 STRING v10: protein-protein interaction networks, integrated over the tree of life. Nucleic Acids Res 43: D447–D452. 25352553 10.1093/nar/gku1003 PMC4383874

[PRASADRNA055871C77] Tichon A, Gil N, Lubelsky Y, Solomon TH, Lemze D, Itzkovitz S, Stern-Ginossar N, Ulitsky I. 2015 A conserved abundant cytoplasmic long noncoding RNA modulates repression by Pumilio proteins in human cells. bioRxiv 10.1101/033423. PMC4947167 27406171

[PRASADRNA055871C78] Uren PJ, Bahrami-Samani E, Burns SC, Qiao M, Karginov FV, Hodges E, Hannon GJ, Sanford JR, Penalva LO, Smith AD. 2012 Site identification in high-throughput RNA-protein interaction data. Bioinformatics 28: 3013–3020. 23024010 10.1093/bioinformatics/bts569 PMC3509493

[PRASADRNA055871C79] Van Etten J, Schagat TL, Hrit J, Weidmann CA, Brumbaugh J, Coon JJ, Goldstrohm AC. 2012 Human Pumilio proteins recruit multiple deadenylases to efficiently repress messenger RNAs. J Biol Chem 287: 36370–36383. 22955276 10.1074/jbc.M112.373522 PMC3476303

[PRASADRNA055871C80] Vessey JP, Schoderboeck L, Gingl E, Luzi E, Riefler J, Di Leva F, Karra D, Thomas S, Kiebler MA, Macchi P. 2010 Mammalian Pumilio 2 regulates dendrite morphogenesis and synaptic function. Proc Natl Acad Sci 107: 3222–3227. 20133610 10.1073/pnas.0907128107 PMC2840302

[PRASADRNA055871C81] Vessey JP, Amadei G, Burns SE, Kiebler MA, Kaplan DR, Miller FD. 2012 An asymmetrically localized Staufen2-dependent RNA complex regulates maintenance of mammalian neural stem cells. Cell Stem Cell 11: 517–528. 22902294 10.1016/j.stem.2012.06.010

[PRASADRNA055871C82] Voronina E, Paix A, Seydoux G. 2012 The P granule component PGL-1 promotes the localization and silencing activity of the PUF protein FBF-2 in germline stem cells. Development 139: 3732–3740. 22991439 10.1242/dev.083980 PMC3445306

[PRASADRNA055871C83] Wang X, Zamore PD, Hall TM. 2001 Crystal structure of a Pumilio homology domain. Mol Cell 7: 855–865. 11336708 10.1016/s1097-2765(01)00229-5

[PRASADRNA055871C84] Wang X, McLachlan J, Zamore PD, Hall TM. 2002 Modular recognition of RNA by a human pumilio-homology domain. Cell 110: 501–512. 12202039 10.1016/s0092-8674(02)00873-5

[PRASADRNA055871C85] Wang Y, Opperman L, Wickens M, Hall TM. 2009 Structural basis for specific recognition of multiple mRNA targets by a PUF regulatory protein. Proc Natl Acad Sci 106: 20186–20191. 19901328 10.1073/pnas.0812076106 PMC2787170

[PRASADRNA055871C86] Weidmann CA, Goldstrohm AC. 2012 *Drosophila* Pumilio protein contains multiple autonomous repression domains that regulate mRNAs independently of Nanos and brain tumor. Mol Cell Biol 32: 527–540. 22064486 10.1128/MCB.06052-11 PMC3255780

[PRASADRNA055871C87] Weidmann CA, Raynard NA, Blewett NH, Van Etten J, Goldstrohm AC. 2014 The RNA binding domain of Pumilio antagonizes poly-adenosine binding protein and accelerates deadenylation. RNA 20: 1298–1319. 24942623 10.1261/rna.046029.114 PMC4105754

[PRASADRNA055871C88] Weyn-Vanhentenryck SM, Mele A, Yan Q, Sun S, Farny N, Zhang Z, Xue C, Herre M, Silver PA, Zhang MQ, 2014 HITS-CLIP and integrative modeling define the Rbfox splicing-regulatory network linked to brain development and autism. Cell Rep 6: 1139–1152. 24613350 10.1016/j.celrep.2014.02.005 PMC3992522

[PRASADRNA055871C89] Wickens M, Bernstein DS, Kimble J, Parker R. 2002 A PUF family portrait: 3′UTR regulation as a way of life. Trends Genet 18: 150–157. 11858839 10.1016/s0168-9525(01)02616-6

[PRASADRNA055871C90] Wilinski D, Qiu C, Lapointe CP, Nevil M, Campbell ZT, Tanaka Hall TM, Wickens M. 2015 RNA regulatory networks diversified through curvature of the PUF protein scaffold. Nat Commun 6: 8213. 26364903 10.1038/ncomms9213 PMC4570272

[PRASADRNA055871C91] Zhang C, Darnell RB. 2011 Mapping *in vivo* protein-RNA interactions at single-nucleotide resolution from HITS-CLIP data. Nat Biotechnol 29: 607–614. 21633356 10.1038/nbt.1873 PMC3400429

[PRASADRNA055871C92] Zhang B, Gallegos M, Puoti A, Durkin E, Fields S, Kimble J, Wickens MP. 1997 A conserved RNA-binding protein that regulates sexual fates in the *C. elegans* hermaphrodite germ line. Nature 390: 477–484. 9393998 10.1038/37297

[PRASADRNA055871C93] Zhu D, Stumpf CR, Krahn JM, Wickens M, Hall TM. 2009 A 5′ cytosine binding pocket in Puf3p specifies regulation of mitochondrial mRNAs. Proc Natl Acad Sci 106: 20192–20197. 19918084 10.1073/pnas.0812079106 PMC2787145

[PRASADRNA055871C94] Zund D, Gruber AR, Zavolan M, Muhlemann O. 2013 Translation-dependent displacement of UPF1 from coding sequences causes its enrichment in 3′ UTRs. Nat Struct Mol Biol 20: 936–943. 23832275 10.1038/nsmb.2635

