# Supplementary material for: The PUF binding landscape in metazoan germ cells
Source: RNA. 2016 Jul;22(7):1026–43. doi: 10.1261/rna.055871.116 (PMC4911911; doi:10.1261/rna.055871.116)
Supplement: Supplemental Material [file supp_22_7_1026__index.html]

Supplemental Material 

# The PUF binding landscape in metazoan germ cells

## Supplemental Material

**Files in this Data Supplement:**

- Supp Fig S1.pdf
- Supp Fig S10.pdf
- Supp Fig S11.pdf
- Supp Fig S12.pdf
- Supp Fig S2.pdf
- Supp Fig S3.pdf
- Supp Fig S4.pdf
- Supp Fig S5.pdf
- Supp Fig S6.pdf
- Supp Fig S7.pdf
- Supp Fig S8.pdf
- Supp Fig S9.pdf
- Supp Table S1.xlsx
- Supp Table S2.xls
- Supp Table S3.xlsx
- Supp Table S4.xls
- Supp Table S5.xlsx
- Supp Text & Legends.docx
