# Supplementary figures and images for: The PUF binding landscape in metazoan germ cells
Source: RNA. 2016 Jul;22(7):1026–43. doi: 10.1261/rna.055871.116 (PMC4911911; doi:10.1261/rna.055871.116)

Figure S1

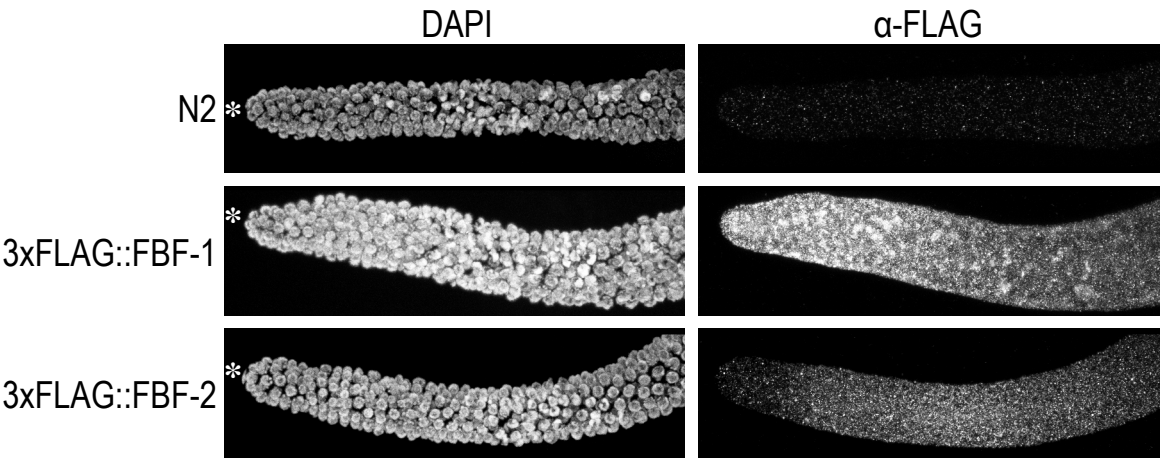

Supplement: Supplemental Material [file supp_055871.116_Supplemental_Fig_S1.pdf]

Figure S10

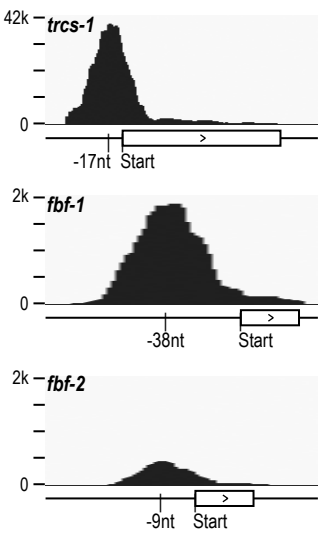

Supplement: Supplemental Material [file supp_055871.116_Supplemental_Fig_S10.pdf]

Figure S11

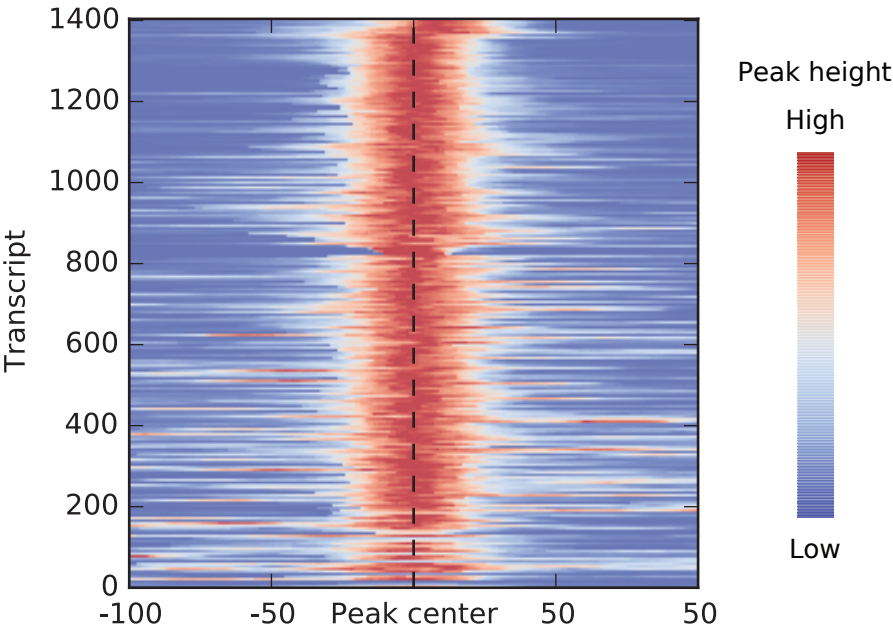

Supplement: Supplemental Material [file supp_055871.116_Supplemental_Fig_S11.pdf]

Figure S2

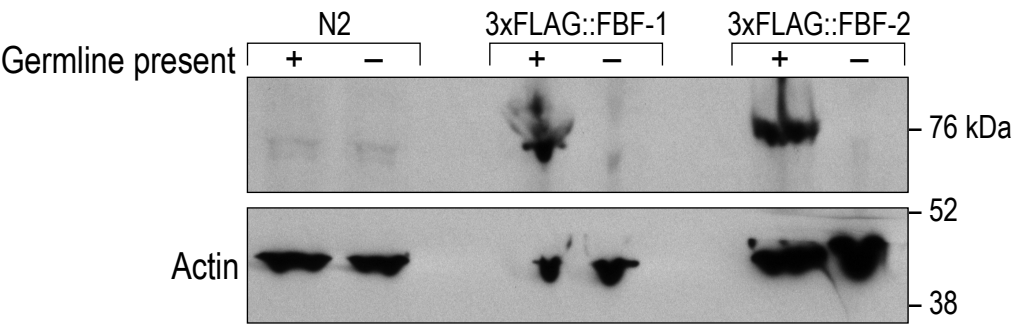

Supplement: Supplemental Material [file supp_055871.116_Supplemental_Fig_S2.pdf]

Figure S5

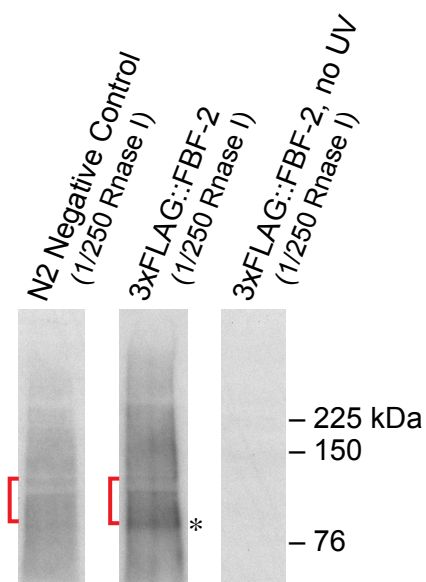

Supplement: Supplemental Material [file supp_055871.116_Supplemental_Fig_S5.pdf]

Figure S8

A. For the canonical FBE

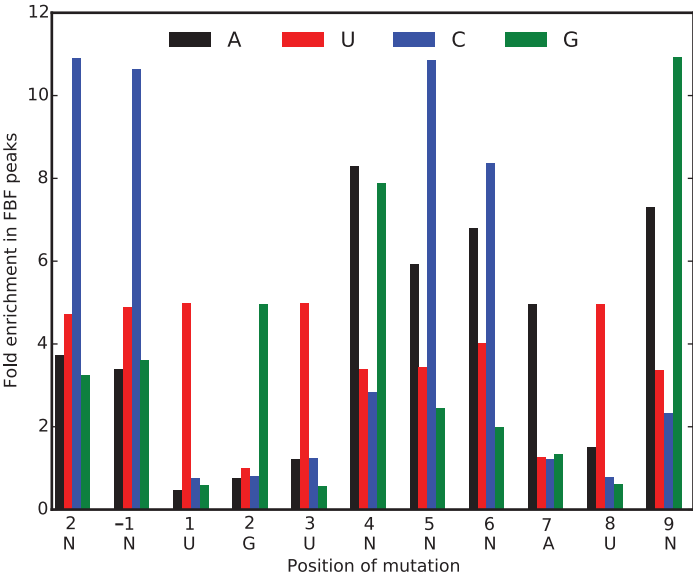

B. For the 7-mer

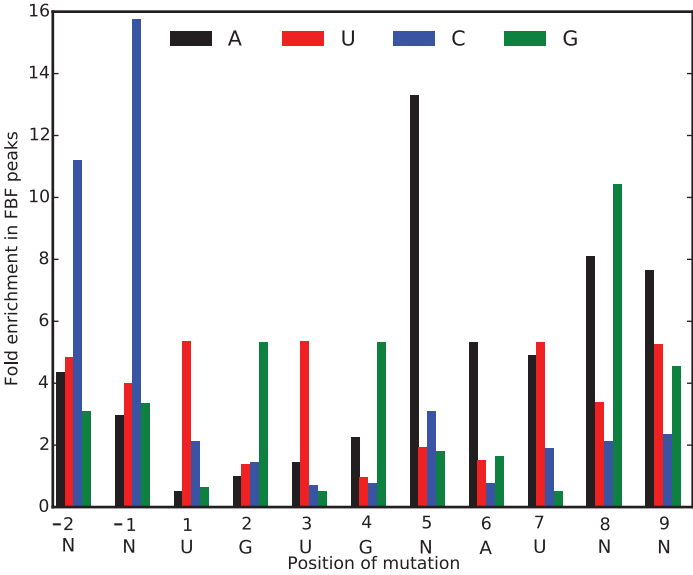

Supplement: Supplemental Material [file supp_055871.116_Supplemental_Fig_S8.pdf]

Figure S9

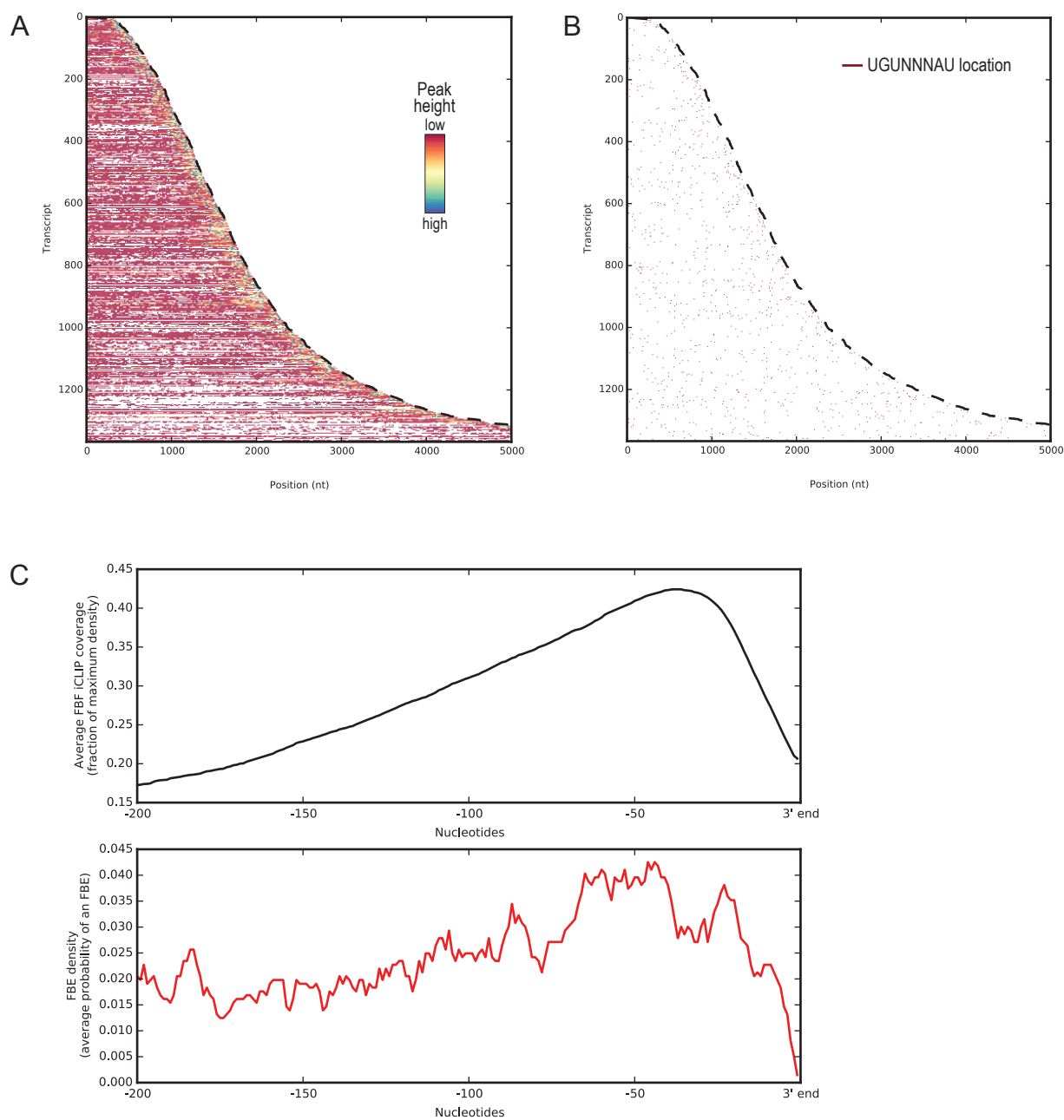

Supplement: Supplemental Material [file supp_055871.116_Supplemental_Fig_S9.pdf]
