# Supplementary material for: The PUF binding landscape in metazoan germ cells
Source: RNA. 2016 Jul;22(7):1026–43. doi: 10.1261/rna.055871.116 (PMC4911911; doi:10.1261/rna.055871.116)
Supplement: Supplemental Material [file supp_055871.116_Supplemental_Fig_S12.pdf]

FBF Targets: Gene Ontology Functional Enrichment Analysis

A

Biological Process	Number of Genes	Bonferroni *P* value
Cell cycle	138	3.1×10^{-57}
Cell cycle process	127	1.0×10^{-52}
Cell cycle phase	110	5.0×10^{-48}
M phase	108	9.7×10^{-47}
Embryonic development	415	1.4×10^{-43}
Meiotic cell cycle	79	9.1×10^{-36}
Meiosis	76	1.9×10^{-34}
M phase of meiotic cell cycle	76	3.3×10^{-34}
Chromosome segregation	71	4.4×10^{-32}
Cell division	74	2.9×10^{-26}

B

Cell Component	Number of Genes	Bonferroni *P* value
Chromosome	37	2.9×10^{-21}
Intracellular non-membrane bounded organelle	75	2.9×10^{-20}
Non-membrane bounded organelle	75	2.9×10^{-20}
Microtubule cytoskeleton	24	5.0×10^{-12}
Cytoskeletal part	27	1.3×10^{-10}
Chromosomal part	21	5.0×10^{-10}
Cytoskeleton	33	3.2×10^{-8}
Microtubule	16	2.5×10^{-7}
Centrosome	9	7.7×10^{-7}
Chromatin	14	3.9×10^{-6}

C

Molecular Function	Number of Genes	Bonferroni *P* value
ATP Binding	118	3.5×10^{-7}
Adenyl ribonucleotide binding	118	2.0×10^{-7}
Purine ribonucleotide binding	132	4.8×10^{-7}
Ribonucleotide binding	132	4.8×10^{-7}
Nucleoside binding	120	2.4×10^{-6}
Adenyl nucleotide binding	119	2.4×10^{-6}
Purine nucleoside binding	119	2.1×10^{-6}
Nucleotide binding	150	1.8×10^{-6}
Helicase activity	24	3.5×10^{-6}
RNA binding	38	2.1×10^{-4}
