# Supplementary material for: The PUF binding landscape in metazoan germ cells
Source: RNA. 2016 Jul;22(7):1026–43. doi: 10.1261/rna.055871.116 (PMC4911911; doi:10.1261/rna.055871.116)
Supplement: Supplemental Material [file supp_055871.116_Supplemental_Fig_S3.pdf]

	Mean mitotic region length (DAPI)	Mean total α-PH3 (+) cells
N2 wild-type n=50 germlines	19.2 ± 2.0	5.8 ± 2.4
FBF-1 transgene n=50	19.8 ± 2.5	4.4 ± 1.4
FBF-2 transgene n=50	20.8 ± 1.8	5.3 ± 2.8
