# Supplementary material for: The PUF binding landscape in metazoan germ cells
Source: RNA. 2016 Jul;22(7):1026–43. doi: 10.1261/rna.055871.116 (PMC4911911; doi:10.1261/rna.055871.116)
Supplement: Supplemental Material [file supp_055871.116_Supplemental_Fig_S4.pdf]

Figure S4

FBF-1 IP-MS	
**Detected Peptide**	**Specificity (* denotes FBF-1 or FBF-2)**
MIETLAHLR	FBF-1 only
TSSFIQSDTDSSRLEPDDFSQNVR	FBF-1 only
SFQSFPNVFMPVSR	*
SNNVLPTWSLDSNGEMR	*
HQQNHIVTSRPPTPLTDLMSLR	*

FBF-2 IP-MS	
**Detected Peptide**	**Specificity**
CFSADIDR	FBF-2 only
CFSADIDRSK	FBF-2 only
EGGYDHAISFQDWLK	FBF-2 only
KMIETLANLR	FBF-2 only
MIETLANLR	FBF-2 only
TSQKPPSTGIDSYPTPAQSPMAQHET PMWDFNSLNPYFSMLNMNDGINYAR	FBF-2 only
TSSFIQSDTDSSRLESDDFSQNVR	FBF-2 only
VVQTIIEKLTADSMNVDLTSAAQNLR	FBF-2 only
AVKGSLTSYQK	*
FASHVVEK	*
FAVDKTGCQFLEK	*
FQLFEQVIGR	*
HQQNHIVTSRPPTPLTDLMSLR	*
LIAICVDQNANHVIQK	*
LMTSVTNR	*
LSLSEVLDSGDLMK	*
LVQALPR	*
NFISSQMTDMCLDKFACR	*
SFQSFPNVFMPVSR	*
SNNVLPTWSLDSNGEMR	*
TSSFIQSDTDSSR	*
VIQSSLQNMDLSLACK	*
VVAVIPLKNWEFIVDFVATPEHLR	*
VVQTIIEK	*
YSRPALSR	*
