# Supplementary material for: The PUF binding landscape in metazoan germ cells
Source: RNA. 2016 Jul;22(7):1026–43. doi: 10.1261/rna.055871.116 (PMC4911911; doi:10.1261/rna.055871.116)
Supplement: Supplemental Material [file supp_055871.116_Supplemental_Fig_S6.pdf]

Figure S6

	Dataset by Replicate	Total Reads	Total Unique Mapped Reads (%)
	**FBF-1 Rep 1**	14,135,062	9,792,191 (69)
	**FBF-1 Rep 2**	5,087,495	3,166,675 (62)
	**FBF-1 Rep 3**	15,000,000	10,408,265 (69)
	**FBF-2 Rep 1**	918,781	884,888 (96)
	**FBF-2 Rep 2**	7,999,348	7,680,463 (96)
	**FBF-2 Rep 3**	5,829,028	5,584,323 (96)
**Controls for FBF-1**	**N2 Rep 1**	12,006,472	7,056,334 (59)
	**N2 Rep 2**	4,424,378	2,476,303 (56)
	**N2 Rep 3**	126,455	63,639 (50)
**Controls for FBF-2**	**N2 Rep 4**	894,145	852,085 (95)
	**N2 Rep 5**	189,420	180,135 (95)
	**N2 Rep 6**	62,701	59,780 (95)
