# Supplementary material for: The PUF binding landscape in metazoan germ cells
Source: RNA. 2016 Jul;22(7):1026–43. doi: 10.1261/rna.055871.116 (PMC4911911; doi:10.1261/rna.055871.116)
Supplement: Supplemental Material [file supp_055871.116_Supplemental_Fig_S7.pdf]

Figure S7

Gene	Biological function	Key Reference
fbf-1	GSC Self-renewal	Lamont et al, 2004
fbf-2	GSC Self-renewal	Lamont et al, 2004
fem-3	spermatogenesis	Zhang et al, 1997
fog-1	spermatogenesis	Thompson et al, 2005
fog-3	spermatogenesis	Thompson et al, 2005
gld-1	meiosis	Crittenden et al, 2002
gld-3S	meiosis	Eckmann et al, 2004
him-3	meiosis	Merritt and Seydoux, 2010
htp-1	meiosis	Merritt and Seydoux, 2010
htp-2	meiosis	Merritt and Seydoux, 2010
syp-2	meiosis	Merritt and Seydoux, 2010
syp-3	meiosis	Merritt and Seydoux, 2010
lip-1	inhibitor of Ras/MAPK	Lee et al, 2006
mpk-1	Ras/MAPK signaling	Lee et al, 2007
egl-4	chemosensation	Kaye et al, 2009
