# Supplementary material for: The PUF binding landscape in metazoan germ cells
Source: RNA. 2016 Jul;22(7):1026–43. doi: 10.1261/rna.055871.116 (PMC4911911; doi:10.1261/rna.055871.116)
Supplement: Supplemental Material [file supp_055871.116_SuppText_Legends.docx]

**Additional data files**

Table S1 presents all detectable peptides from IP-MS analysis. Specifically, tab 1 is analysis of 3xFLAG::FBF-1 and tab 2 is analysis of 3xFLAG::FBF-2. Table S2 presents iCLIP peak data for FBF-1, FBF-2, and “FBF targets”. Columns in the table list peak heights as raw values and also as values normalized to RNA abundance; for the latter, we report normalization to either of two RNA-seq datasets (whole worm or whole gonad). Specifically, tab 1 is FBF-1 versus negative control for FBF-1, tab 2 is FBF-1 versus negative control for FBF-2, tab 3 is FBF-2 versus negative control for FBF-2, tab 4 and tab 5 are “FBF peaks” with and without non-coding RNA, and tab 6 and tab 7 are “FBF peaks” present and not present in a previous RIP-chip analysis (Kershner and Kimble 2010). Table S3 presents target genes two-fold enriched (log scale) by read count in either FBF-1 or FBF-2 datasets. Table S4 presents FBF targets by peak location. Specifically, tab 1 is targets that contained at least one 5’UTR peak, tab 2 is targets containing at least one coding sequence (CDS) peak, tab 3 is targets containing 3’UTR and 5’UTR peaks, tab 4 is targets containing 3’UTR and CDS peaks, and tab 5 is targets containing peaks in all three locations. Table S5 presents tab 1: FBF targets critical for germline sex determination, tab 2: FBF targets shared with yeast Puf3 and Puf5, tab 3: FBF targets shared with human PUM2, tab 4: FBF targets shared with yeast Puf3, Puf5, and human PUM2, tab 5: FBF targets that have human orthologues, tab 6: FBF cell cycle related targets are also human PUM2 targets.

**SUPPLEMENTAL FIGURES**

**Figure S1.** Germline distribution of FLAG-tagged FBF-1 and FBF-2, assayed by immunocytochemistry of gonads dissected from transgenic animals [FBF-1: *fbf-1(0)* *3xflag::fbf-1* and FBF-2: *fbf-2(0)* *3xflag::fbf-2*]. Asterisk marks the distal end of the gonad. Left, DAPI highlights germ cell nuclei. Right, α-FLAG staining reveals distal enrichment of FLAG-tagged FBF-1 (middle right) and FBF-2 (lower right).

**Figure S2.** FLAG-tagged FBF is abundant in control animals with normal germline tissue, but is not detectable in animals lacking all germline tissue. Worms lacking germline tissue were produced by double RNAi against *sygl-1* and *lst-1* (Kershner et al. 2014). Actin was used as a loading control.

**Figure S3.** The size of the germline mitotic region and number of actively dividing germline cells in FBF-1 and FBF-2 transgenic worms was similar to wild-type. The size of the mitotic region was measured in germ cell diameters. The number of actively dividing cells was assayed as the number of α-PH3 (phosphorylated histone H3)-positive nuclei.

**Figure S4.** IP-mass spectrometry peptide identifications at FDR ≤ 1% for two pooled, UV cross-linked biological replicates of tagged FBF-1 and FBF-2. Asterisks indicate peptides that could be from either FBF-1 or FBF-2, given that their amino acid sequences are 91% identical. Two pooled N2 biological replicates were processed in parallel as negative controls for each FBF. No FBF peptides were detected in control samples.

**Figure S5.** Representative autoradiograph of immunopurified and ^32^P-labeled protein:RNA complexes transferred to nitrocellulose membrane and exposed for 5 hours on film. Lysates were treated with an RNase I concentration of 1/250 resulting in a smear of radioactive signal from protein-RNA complexes of various sizes. The asterisk marks a band corresponding to highly digested complexes near the expected molecular weight mark of 76 kDa. Red markings indicate the approximate region of the membrane that was excised and processed for iCLIP in both experiment and control. Radioactive signal required UV crosslinking (compare middle and right lanes).

**Figure S6.** Table of total and unique (with MAPQ quality score > 20) high-throughput sequencing reads mapped to *C. elegans* genome release WS235. We performed three biological replicates each of FBF-1, FBF-2, and wild-type N2 negative controls. Percentages shown in parentheses are the percent of total reads.

**Figure S7.** Validated targets of FBF-1 and/or FBF-2 supported by multiple lines of evidence. Evidence is documented in the associated reference.

**Figure S8.** **A.** Variations in the FBE are enriched at different levels. Only joint FBF-1 and FBF-2 peaks (5/6 replicates) were included in this analysis. Peak regions were defined as a 70 nt region around the highest point of coverage. Background for a given peak was defined as the average of 1000 random permutations of the 70 nt sequence. Enrichment over the randomized sequences was calculated for each mutation in the FBE. Unlike previous *in vitro* studies, we observe a preference for purine at position 10 and find that a C at positions 5 and 6 is strongly favored *in vivo*. **B.** Similar analysis as in panel **A** for the 7-mer enriched in peaks without a canonical FBE.

**Figure S9. A and B.** Heat maps of peak signal (**A**) and the canonical FBE sequence (**B**) in the mature transcripts of target mRNAs. As in Figure 7E, for all peaks FBF binding is biased toward the 3’-most end. **C.** Plots of 3’ proximity of FBEs and average iCLIP coverage relative to the end of 3’UTRs in FBF target mRNAs.

**Figure S10.** Representative snapshots of FBF binding in the 5’UTR of select targets, with distance to the start codon indicated. The y-axis denotes total coverage depth from FBF-1 and FBF-2.

**Figure S11.** Heat map aligned on the primary peak for each FBF target indicates a characteristic shape for primary peaks: a sharp, nearly symmetrical region roughly 40 nucleotides wide.

**Figure S12.** Top 10 enriched Gene Ontology terms for the categories of (**A**) Biological Process, (**B**) Cell Component, and (**C**) Molecular Function by DAVID analysis.
